# Whole-genome analysis of carbapenem-resistant *Acinetobacter baumannii* from clinical isolates in Southern Thailand

**DOI:** 10.1016/j.csbj.2021.12.038

**Published:** 2022-01-06

**Authors:** Arnon Chukamnerd, Kamonnut Singkhamanan, Virasakdi Chongsuvivatwong, Prasit Palittapongarnpim, Yohei Doi, Rattanaruji Pomwised, Chanida Sakunrang, Kongpop Jeenkeawpiam, Mingkwan Yingkajorn, Sarunyou Chusri, Komwit Surachat

**Affiliations:** a Department of Biomedical Sciences and Biomedical Engineering, Faculty of Medicine, Prince of Songkla University, Songkhla, Thailand; b Epidemiology Unit, Faculty of Medicine, Prince of Songkla University, Songkhla, Thailand; c Pornchai Matangkasombut Center for Microbial Genomics, Department of Microbiology, Faculty of Science, Mahidol University, Bangkok, Thailand; d Division of Infectious Diseases, University of Pittsburgh School of Medicine, Pittsburgh, PA, USA; e Department of Microbiology, Fujita Health University, Aichi, Japan; f Division of Biological Science, Faculty of Science, Prince of Songkla University, Songkhla, Thailand; g Molecular Evolution and Computational Biology Research Unit, Faculty of Science, Prince of Songkla University, Songkhla, Thailand; h Department of Pathology, Faculty of Medicine, Prince of Songkla University, Songkhla, Thailand; i Division of Infectious Diseases, Department of Internal Medicine, Faculty of Medicine, Prince of Songkla University, Songkhla, Thailand; j Division of Computational Science, Faculty of Science, Prince of Songkla University, Songkhla, Thailand

**Keywords:** Carbapenem-resistant *Acinetobacter baumannii* (CRAB), Whole-genome sequencing (WGS), Bioinformatics tool, Sequence type (ST), Antimicrobial resistance (AMR) gene, Mobile genetic element (MGE)

## Abstract

The worldwide spread of carbapenem-resistant *Acinetobacter baumannii* (CRAB) has become a healthcare challenge for some decades. To understand its molecular epidemiology in Southern Thailand, we conducted whole-genome sequencing (WGS) of 221 CRAB clinical isolates. A comprehensive bioinformatics analysis was performed using several tools to assemble, annotate, and identify sequence types (STs), antimicrobial resistance (AMR) genes, mobile genetic elements (MGEs), and virulence genes. ST2 was the most prevalent ST in the CRAB isolates. For the detection of AMR genes, almost all CRAB isolates carried the *bla*_OXA-23_ gene, while certain isolates harbored the *bla*_NDM-1_ or *bla*_IMP-14_ genes. Also, various AMR genes were observed in these CRAB isolates, particularly aminoglycoside resistance genes (e.g., *armA*, *aph(6)-Id*, and *aph(3″)-Ib*), fosfomycin resistance gene (*abaF*), and tetracycline resistance genes (*tet*(B) and *tet*(39)). For plasmid replicon typing, RepAci1 and RepAci7 were the predominant replicons found in the CRAB isolates. Many genes encoding for virulence factors such as the *ompA*, *adeF*, *pgaA*, *lpxA*, and *bfmR* genes were also identified in all CRAB isolates. In conclusion, most CRAB isolates contained a mixture of AMR genes, MGEs, and virulence genes. This study provides significant information about the genetic determinants of CRAB clinical isolates that could assist the development of strategies for improved control and treatment of these infections.

## Graphical abstract

**Figure:**
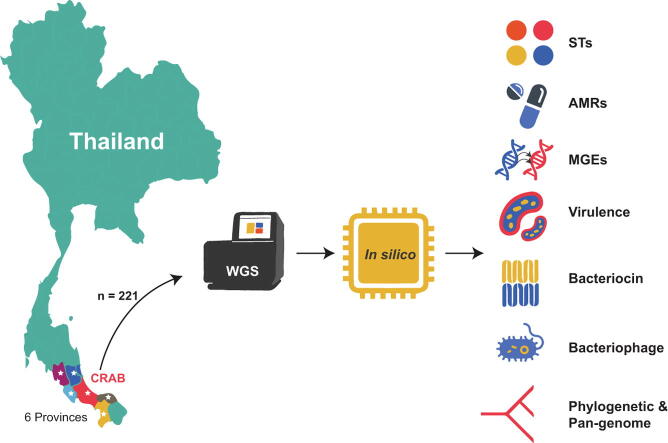

## 1 Introduction

Multidrug resistance (MDR) in Gram-negative bacteria is a global public health concern as the treatment options are dramatically limited [1], [2]. These pathogens have a high level of resistance to available antimicrobial classes, especially carbapenems and colistin, which are considered to be the last-line treatments [3], [4], [5]. Among them, carbapenem-resistant *Acinetobacter baumannii* (CRAB) is an important cause of nosocomial infections associated with high mortality rates [6]. It is commonly transmitted in intensive care units (ICUs). CRAB can cause various infections such as ventilator-associated pneumonia, wound infections, urinary tract infections (UTIs), bloodstream infections, and meningitis [7], [8].

CRAB can be resistant to carbapenems through various mechanisms. Carbapenemase production is the major mechanism of carbapenem resistance in *A. baumannii* as well as other Gram-negative bacteria. The carbapenemase enzymes have been classified by Ambler into three classes, class A, class B, and class D carbapenemases [9]. The carbapenemase-encoding genes are mainly located on mobile genetic elements (MGEs), plasmids, transposons, and integrons. Due to the presence of MGEs, many carbapenemase genes can be transferred between plasmids and chromosomes. Additionally, they can be horizontally transferred from one bacteria to another bacteria, leading to the rapid dissemination of carbapenemase genes [10]. Although the genetic basis associated with antimicrobial resistance (AMR) and bacterial pathogenesis among CRAB isolates has been characterized [11], [12]. The genetic processes supporting the co-acquisition of multiple carbapenemase genes as well as other AMR genes still need to be elucidated further. Whole-genome sequencing (WGS) has become a powerful tool for rapidly analyzing the entire genomic DNA sequence of organisms. It has been used to characterize and understand the mechanisms of AMR and their spread through bacterial species, which is necessary for combating AMR-bacteria [13], [14].

Previously, most of the studies have been reported the genetic characteristics of CRAB clinical isolates from many countries such as Korea, Thailand, Vietnam, Myanmar, Malaysia, and Brazil as well as in European countries [1], [3], [15], [16], [17], [18]. Their findings demonstrated that a high level of the CRAB isolates was assigned to an ST2 with the carriage of the *bla*_OXA-23_ gene and other AMR genes conferring resistance to many antibiotic classes, particularly aminoglycosides. In addition, the RecAci1 plasmid was predominantly found in the CRAB isolates, while the insertion sequence (IS) elements (e.g., IS*Aba1* and IS*Aba125*) were also detected in the CRAB isolates. Although several studies have provided WGS data of many CRAB isolates which are for understanding the distribution of AMR genes, virulence genes, and MGEs, there are to date few studies examining the genomic characteristics of CRAB isolates from Southern Thailand. Elucidation of the mechanisms of acquired AMR genes and virulence-associated genes and the genomic diversity among the CRAB isolates would help better understand their dissemination patterns in the regions. This is because CRAB isolates continuously evolve to survive in harsh environments, and they have been spreading throughout the world for a long time. Importantly, the AMR genes can be horizontally transferred (conjugation and transduction) to other related pathogenic bacteria, causing the rapid and global spread of AMR in Gram-negative bacteria. Thus, the objective of this study was to analyze the whole-genome sequence of CRAB, isolated from patients in 7 hospitals within lower Southern Thailand, to gain genomic insights into the clinical CRAB isolates of this area. The understanding and tracing of the rapid evolution of MDR in Gram-negative bacteria will play an important role in controlling these bacteria and slowing their spread until more effective treatments become available.

## 2 Materials and methods

### 2.1 Bacterial isolates and clinical data

**Fig. 1:**
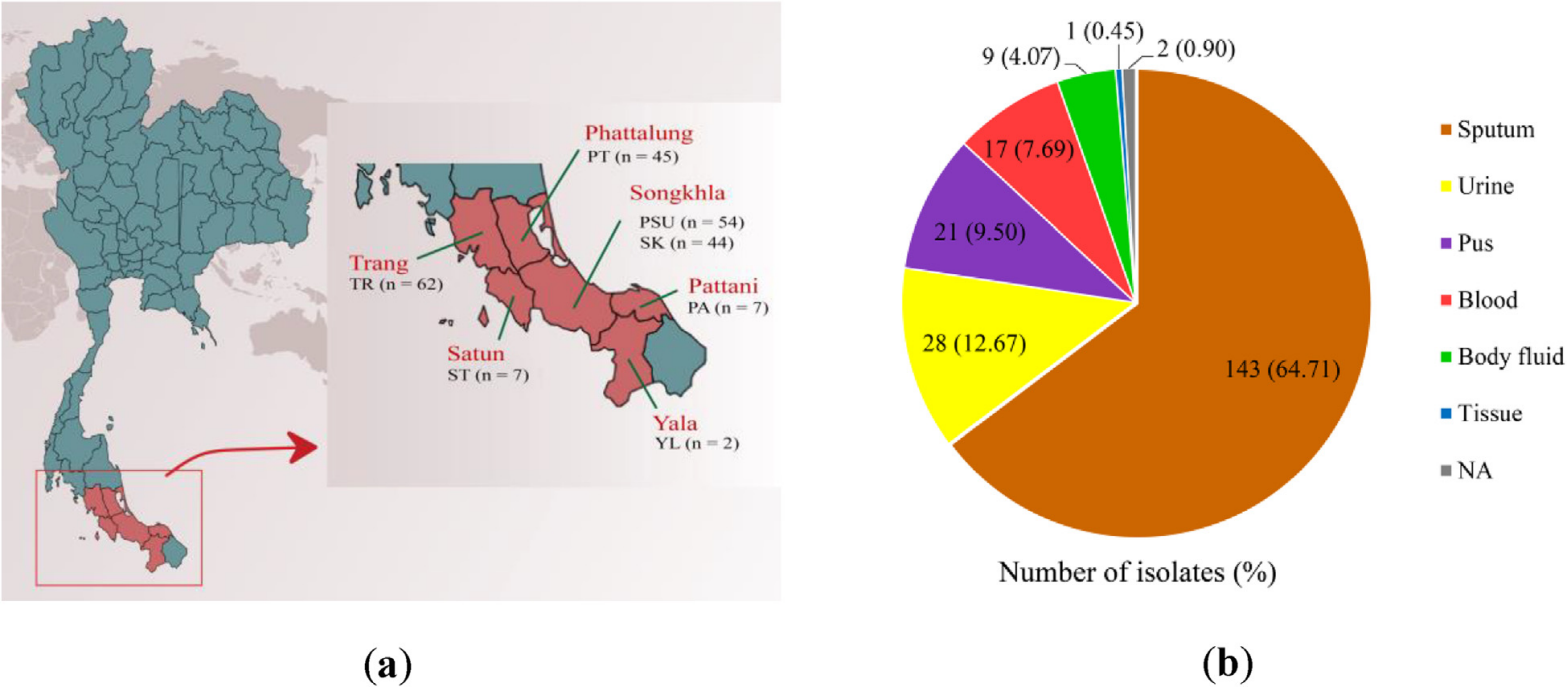
The numbers of CRAB clinical isolates collected at hospitals located in 6 provinces, lower Southern Thailand (a) and sample sources (b). TR, Trang Hospital; PSU, Songklanagarind Hospital; PT, Phatthalung Hospital; SK, Songkhla Hospital; ST, Satun Hospital; PA, Pattani Hospital; YL, Yala Hospital; NA, not available.

In this study, a total of 221 CRAB isolates were obtained from the Clinical Microbiology Laboratories (CMLs) of 7 hospitals located in lower Southern Thailand including Trang Hospital (n = 62; 28.05%), Songklanagarind Hospital (n = 54; 24.43%), Phatthalung Hospital (n = 45; 20.36%), Songkhla Hospital (n = 44; 19.91%), Satun Hospital (n = 7; 3.17%), Pattani Hospital (n = 7; 3.17%), and Yala Hospital (n = 2; 0.90%) (Fig. 1a). The 221 CRAB isolates were collected mostly from sputum but also from other clinical specimens (e.g., urine, pus, blood, body fluids, and tissue) of 221 patients who were admitted to the hospitals between March and August 2019 (Fig. 1b). In CMLs, the *A. baumannii* strains were identified by biochemical tests, according to Bergey's Manual of Systematic Bacteriology [19] and confirmed by Matrix-Assisted Laser Desorption/Ionization-Time Of Flight (MALDI-TOF) mass spectrometry (MS) [20], [21]. The phenotypic resistance to carbapenem (imipenem and meropenem) in *A. baumannii* strains was evaluated by disk diffusion method. *A. baumannii* strains were defined as resistant to carbapenem, when the zone diameters were ≤ 18 mm for imipenem and/or ≤ 14 mm for meropenem, according to the CLSI guideline (2018) [22]. The inclusion criterion for CRAB isolates was the *A. baumannii* strains that were resistant to carbapenem, while *A. baumannii* strains without carbapenem resistance were excluded from the study.

### 2.2 Genome library preparation and sequencing

Genomic DNA of all the CRAB isolates was extracted using the TIANamp Bacterial DNA Kit (Tiangen, Beijing, China), following the manufacturer’s instructions. The extracted DNA was sent to the Beijing Genomics Institute (BGI) in China for short-read WGS. For testing sample qualification, the DNA concentrations were measured by Qubit Fluorometer (Invitrogen), while DNA integrity and purity were investigated by Agarose Gel Electrophoresis. Then, 1 µg of the qualified genomic DNA (≥23 kbp) was randomly fragmented by Covaris. Fragmented sequences with a size of ≤ 800 bp were selected using the Agencourt AMPure XP-Medium kit. End-repair and 3′-adenylation were performed on the fragments, and adaptors were ligated to the ends of these 3′-adenylated fragments to amplify the fragments. The PCR products were then purified using an Agencourt AMPure XP-Medium kit. The double-stranded PCR products were heat-denatured and circularized by the splint oligo sequence. The single-strand circle DNA (ssCir DNA) was formatted as the final library. The quality of the whole-genome library was checked by quality control (QC). The qualified libraries were sequenced by BGISEQ-500 (BGI, China). Finally, 150-bp paired-end reads were received by combinatorial Probe-Anchor Synthesis (cPAS).

### 2.3 Genome assembly and annotation

*De novo* assemblies of our 221 CRAB genomes were generated using SPAdes v3.12 [23]. The quality and completeness of the genome assemblies were assessed by Quast v5.0.2 [24] and Busco v5.1.2 [25], [26], respectively. According to the exploration of reported *A. baumannii* genomes in the National Center for Biotechnology Information (NCBI) (*https://www.ncbi.nlm.nih.gov/*), we predicted that the highest length of *A. baumannii* genomes is approximate 4.4 Mbp. Thus, assembled sequences containing a read length of > 4.4 Mbp were initially excluded from the study, because they might contain contaminant sequences from other species. The genomes were then annotated using Prokka v1.12 [27], and finally, the tRNAs and rRNAs were identified by tRNAscan-SE v2.0 [28], [29] and RNAmmer v1.2 [30], respectively.

### 2.4 Bioinformatics analysis

Sequence analyses were performed using several bioinformatics tools. Although the CMLs of the 7 hospitals had previously identified the CRAB isolates using standard biochemical methods and MALDI-TOF MS, the *A. baumannii* species was reconfirmed by *in silico* methods using SpeciesFinder v2.0 (*https://cge.cbs.dtu.dk/services/SpeciesFinder/*) [31] in the center for genomic epidemiology (CGE). For multilocus sequence typing (MLST), we searched the sequence types (STs) of all CRAB isolates against the public databases for molecular typing and microbial genome diversity (PubMLST) using mlst v2.19.0 (*https://github.com/tseemann/mlst*) [32]. The AMR genes were identified using ABRicate v1.0.1 (*https://github.com/tseemann/abricate*) with the default parameter against the comprehensive antibiotic resistance database (CARD) (*https://card.mcmaster.ca/*) [33]. In plasmid identification, we created a plasmid nucleotide sequence database based on the literature reviews [16], [34], [35], [36], [37], [38], [39], [40], [41], [42] and then predicted the presence of plasmid replicon types using blastn v2.12.0 with 80% identity and 1e-30 E-value cut-offs. The presence of insertion sequence (IS) elements was predicted using ABRicate v1.0.1 with the default parameter against the IS database from ISfinder (*https://www-is.biotoul.fr/*) [43]. Also, the integrons were investigated using integron_finder v2.0 (*https://github.com/gem-pasteur/Integron_Finder*) with the default parameter [44]. Also, the virulence-associated genes were detected using blastn v2.12.0 with 80% identity and 1e-30 E-value cut-offs against the virulence factor database (VFDB) of *Acinetobacter* spp. (http://www.mgc.ac.cn/cgi-bin/VFs/genus.cgi?Genus=Acinetobacter) [45], while the bacteriocin-encoding genes were explored using blastx v2.12.0 with 80% identity and 1e-30 E-value cut-offs against the databases from bacteriocin genome mining tool (BAGEL4) (*http://bagel4.molgenrug.nl/databases.php*) [46]. We also predicted the presence of the bacteriophage genome in the CRAB isolates using phigaro v2.3.0 (*https://github.com/bobeobibo/phigaro*) with the default parameter [47].

### 2.5 Pan-genome and phylogenetic analysis

The pan-genome of our 221 CRAB isolates was analyzed using Roary v3.13.0 [48], with a 95% minimum blastp identity and a 99% core definition threshold. Then, we called SNPs of core genes to reduce the computational complexity for phylogenetic tree construction using SNP-sites v2.4.1 [49]. Afterward, a phylogenetic tree was then built by raxmlHPC-PTHREADS v8.2.12 with the neighbor-joining method using 1000 bootstraps [50]. Visualization of the phylogenetic tree was performed using Geneious R10.26 [51] and Phandango website (*https://jameshadfield.github.io/phandango/*) [52]. A pan-genome frequency plot, a piechart of the pan-genome, and a presence and absence matrix against a phylogenetic tree were created using roary_plots script (*https://github.com/sanger-pathogens/Roary/tree/master/contrib/roary_plots*). In addition, we also performed the pan-genome analysis of our CRAB genomes compared to previously published genomes from Thailand [16]. The phylogenetic trees were constructed based on the SNPs of core genes and accessory genes, respectively, with the neighbor-joining method using 1000 bootstraps.

## 3 Results

### 3.1 Patient demographics and clinical profiles

Since we selected only 1 isolate per patient for performing WGS, the prevalence and distribution of patients in each hospital were equal to the number of isolates, as noted earlier. The clinical profiles showed that, in the 221 patients infected with CRAB, diabetes mellitus was the most common underlying disease (n = 103; 46.61%), followed by hypertension (n = 78; 35.29%), chronic kidney disease (n = 48; 21.72%), cerebrovascular disease (n = 42; 19.00%), coronary artery disease (n = 38; 17.19%), and pulmonary disease (n = 29; 13.12%). Importantly, 199 (90.04%) of the 221 patients had previously received carbapenem antibiotics (meropenem, imipenem, and/or ertapenem). We found the prior use of ceftriaxone, piperacillin-tazobactam, fluoroquinolones (levofloxacin or ciprofloxacin), ceftazidime, and aminoglycosides (amikacin or gentamicin) in 139 (62.90%), 116 (52.49%), 77 (34.84%), 27 (12.22%), and 27 (12.22%) patients. The previous use of other antibiotics (azithromycin, colistin, tigecycline, or cefoperazone-sulbactam) was also found in some patients. The metadata of the patients is shown in Table S1.

### 3.2 Genome assembly quality

In the 221 CRAB isolates, *de novo* assembly yielded genome lengths from 3,777,937 bp to 4,319,283 bp, with an average of 3,930,367 bp. The number of contigs ranged from 26 to 193, with an average of 68. The GC-content varied from 38.68% to 39.10%, with an average of 38.91%. The N50 and L50 values of the 221 assembled genomes ranged from 45,197 bp to 444,207 bp having an average of 175,941 bp and 3 to 28 having an average of 9, respectively. The details of the assembly quality are given in Table S2.

### 3.3 Sequence types (STs) and antimicrobial resistance (AMR) determinants

In the MLST results of the 221 CRAB isolates (Table S3), ST2 had the largest frequency having been identified in 119 (53.85%) isolates, followed by ST164 (n = 29; 13.12%), ST374 (n = 18; 8.14%), ST16 (n = 13; 5.88%), ST215 (n = 12; 5.43%), and so forth. However, STs could not be assigned for 2 (0.90%) isolates, PSU043 and PSU114. Among the 7 housekeeping genes of *A. baumannii*, using MLST (Pasteur), the alignment of PSU043 genome with allele 8 of the *recA* gene showed 99.73% identity and 100% coverage. This PSU043 contained 1 nucleotide substitution (A to G) at position 99 of the *recA* gene sequence, resulting in a non-identified ST, while PSU114 provided 100% identity and 100% coverage in alignment with all 7 genes. However, when the locus combination of allele 181 of the *gltA* gene and the alleles of the 6 other genes were analyzed, according to the MLST allelic profile, PSU114 could not be assigned to an ST.

**Fig. 2:**
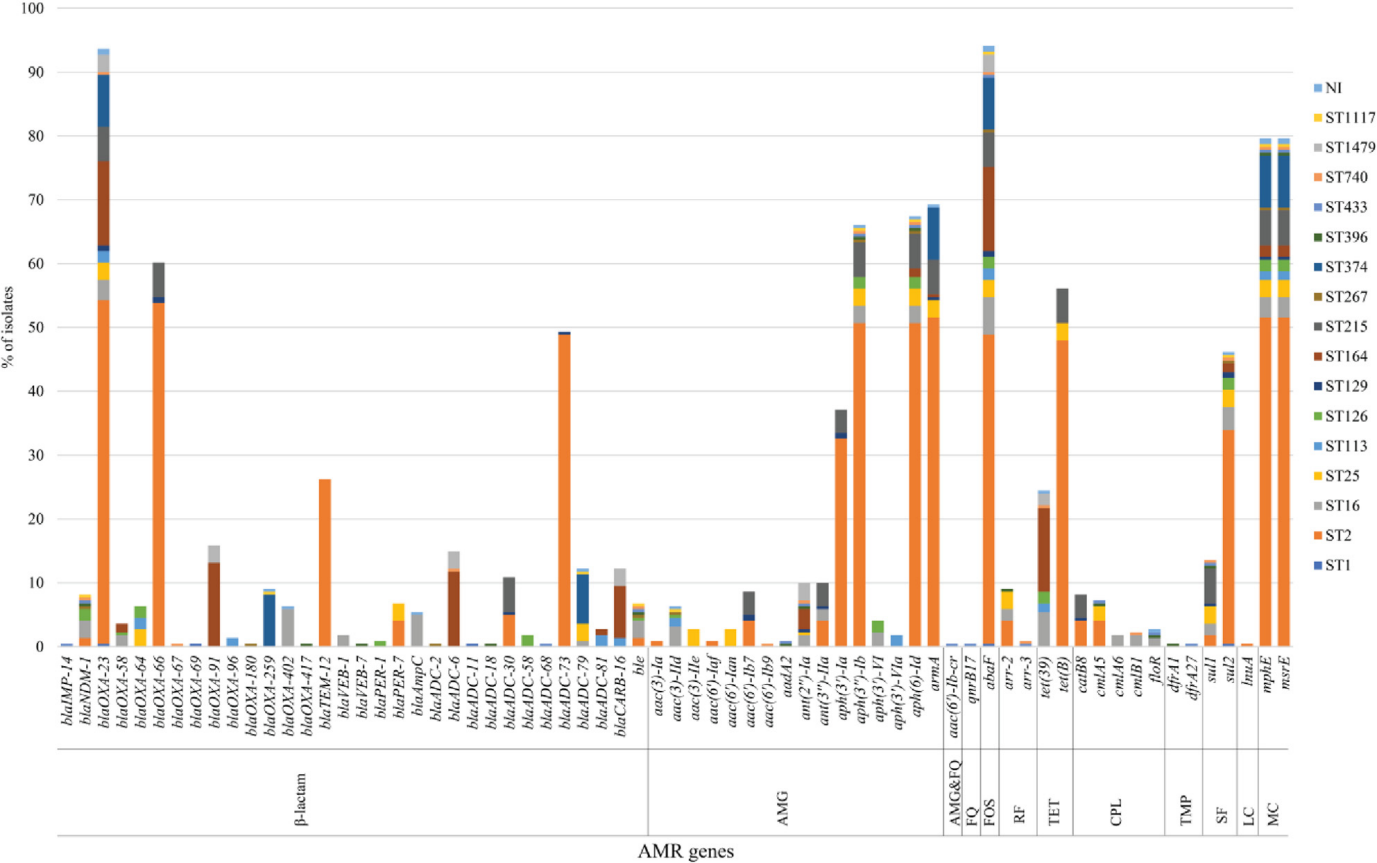
Distribution of antimicrobial resistance (AMR) genes in the study 221 CRAB clinical isolates according to sequence types (STs). NI, non-identified ST; AMG, aminoglycoside; FQ, fluoroquinolone; FOS, fosfomycin; RF, rifampicin; TET, tetracycline; CPL, chloramphenicol; TMP, trimethoprim; SF, sulfonamide; LC, lincosamide; MC, macrolide.

**Fig. 3:**
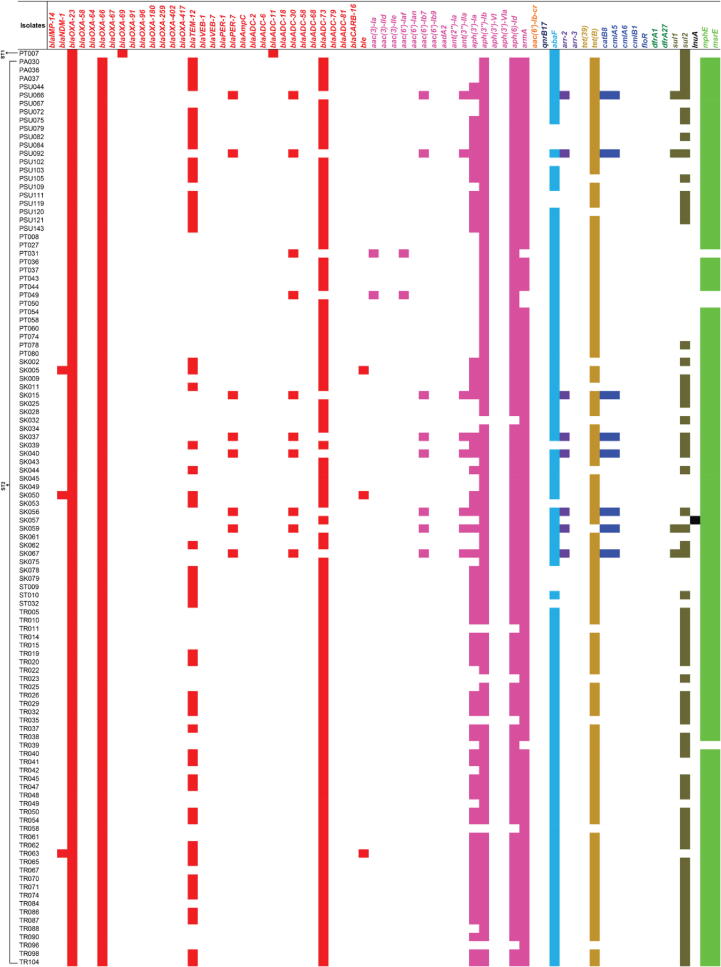

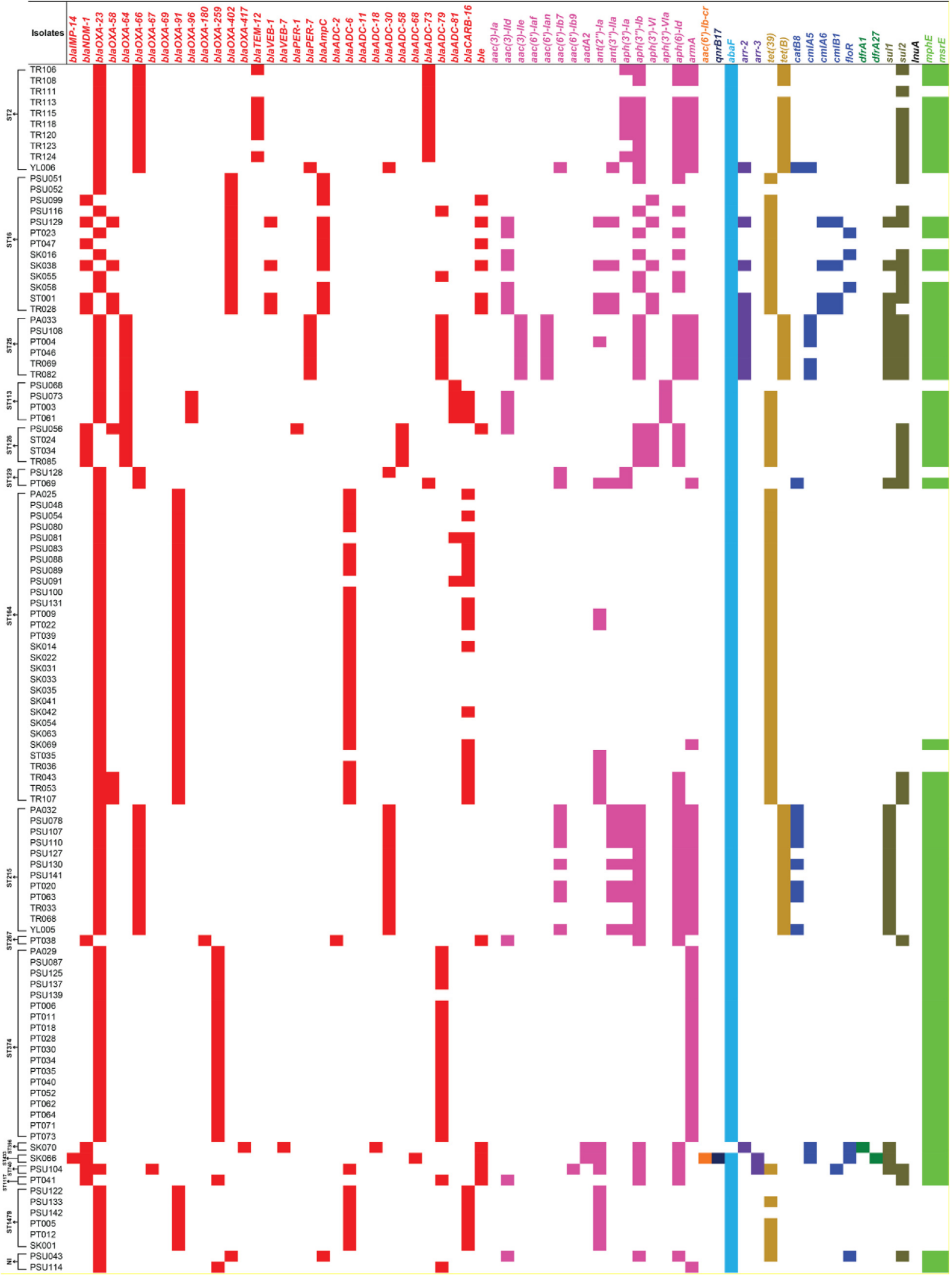
The presence of antimicrobial resistance (AMR) genes in the study 221 CRAB clinical isolates. Red, pink, orange, dark blue, light blue, purple, dark gold, blue, dark green, solid bracken green, black, and green colors represent the predicted resistance to β-lactam, aminoglycoside, aminoglycoside and fluoroquinolone, fluoroquinolone, fosfomycin, rifampicin, tetracycline, chloramphenicol, trimethoprim, sulfonamide, lincosamide, and macrolide, respectively. (For interpretation of the references to color in this figure legend, the reader is referred to the web version of this article.)

Identification of AMR determinants from the WGS data revealed various AMR determinants with the predicted resistance to several antimicrobial classes, as shown in Fig. 2, Fig. 3, Table S4. Among the 221 CRAB isolates, 207 (93.66%) isolates carried the *bla*_OXA-23_ gene, while only 18 (8.14%), 8 (3.62%), and 1 (0.45%) isolates harbored the *bla*_NDM-1_, *bla*_OXA-58_, and *bla*_IMP-14_ genes, respectively. These genes were the carbapenemase genes that might be expressed, leading to carbapenem resistance in these CRAB isolates. Likewise, we found the *bla*_OXA-66_ (n = 133; 60.18%), *bla*_ADC-73_ (n = 109; 49.32%), *bla*_TEM-12_ (n = 58; 26.24%), *bla*_OXA-91_ (n = 35; 15.84%), *bla*_ADC-79_ (n = 27; 12.22%), and *bla*_CARB-16_ (n = 27; 12.22%) genes and so forth, which may provide resistance to other β-lactam antibiotics such as penicillins and cephalosporins. For aminoglycoside resistance prediction, the *armA*, *aph(6)-Id*, and *aph(3″)-Ib* genes were highly detected in 153 (69.23%), 149 (67.42%), and 146 (66.06%) isolates, respectively. Additionally, we found more aminoglycoside resistance genes such as the *aph(3′)-Ia* (n = 82; 37.10%), *ant(2″)-Ia* (n = 22; 9.95%), *ant(3″)-IIa* (n = 22; 9.95%), and *aac(6′)-Ib7* (n = 19; 8.60%) genes, and so forth. Notably, the *abaF* gene that probably provides resistance to fosfomycin was present in 208 (94.12%) isolates. In addition, 176 (79.64%) isolates possessed the *mphE* and *msrE* genes predicting macrolide resistance, while 124 (56.11%) and 54 (24.43%) isolates contained the *tet*(B) and *tet*(39) genes predicting tetracycline resistance. The *sul2* and *sul1* genes that may confer resistance to sulfonamide were observed in 102 (46.15%) and 30 (13.57%) isolates, respectively. We also found other genes such as the *arr-2* (n = 20; 9.05%) and *catB8* (n = 18; 8.14%) genes, which may provide resistance to rifampicin and chloramphenicol, respectively. Besides the investigation of the presence of AMR genes, almost all CRAB isolates might be classified as MDR isolates since they possessed many genes that probably confer resistance to more than three antimicrobial classes.

In terms of the ST distribution of AMR genes in the 221 CRAB isolates, we found that all ST2 isolates harbored the *bla*_OXA-23_ and *bla*_OXA-66_ genes (Fig. 2, Fig. 3, Table S4). ST2 isolates carried various AMR genes, ranging from 5 to 19 in number. In addition, some AMR genes, especially the *aph(3″)-Ib*, *aph(6)-Id*, *armA*, *abaF*, *sul2*, *mphE*, and *msrE* genes were detected in many STs. The *bla*_OXA-69_ and *bla*_ADC-11_ genes were only present in ST1 isolate (PT007), while the *bla*_OXA-417_ and *bla*_VEB-7_ genes were only found in ST396 isolate (SK070). The *bla*_IMP-14_, *bla*_ADC-68_, *aac(6′)-Ib-cr*, *qnrB17*, and *dfrA27* genes were only identified in ST433 isolate (SK066), while the *bla*_OXA-67_ and *aac(6′)-Ib9* genes were only seen in ST740 isolate (PSU104). Also, only ST25 isolates possessed the *aac(3)-IIe* and *aac(6′)-Ian* genes, while only ST113 isolates harbored the *aph(3′)-VIa* gene.

### 3.4 Mobile genetic elements (MGEs)

**Fig. 4:**
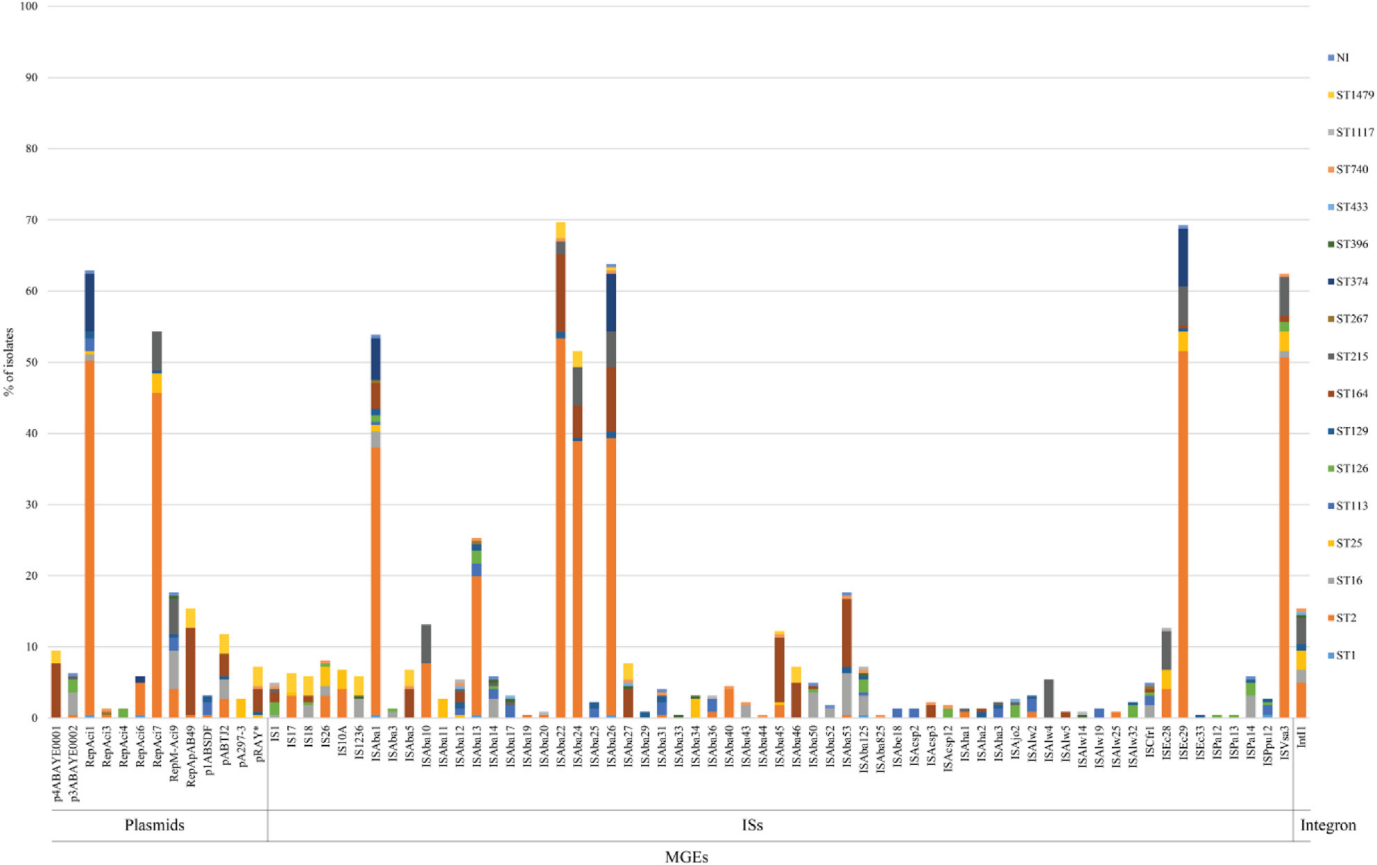
Distribution of mobile-genetic elements (MGEs) in the study 221 CRAB clinical isolates according to sequence types (STs). NI, non-identified ST; ISs, insertion sequences.

Overall, the plasmid replicons were identified in 219 (99.10%) of the CRAB isolates, while no plasmids were found in the other 2 (0.90%) isolates, PT041 and SK066. A total of 169 (76.47%) isolates contained 2 to 6 plasmid types, while 50 (22.62%) isolates carried only one plasmid, as shown in Fig. 4 and S1, Table S5. Notably, PT069 contained the highest number of plasmids, RepAci1, RepAci7, RepM-Aci9, p1ABSDF, pABTJ2, and pRAY*. The RepAci1 and RepAci7 plasmids were detected at a high frequency in 139 (62.90%) and 120 (54.30%) isolates, respectively, and they were present in almost all ST2 isolates. The RepM-Aci9 plasmid was harbored by 39 (17.65%) isolates, followed by RepApAB49 (n = 34; 15.38%), pABTJ2 (n = 26; 11.76%), p4ABAYE0001 (n = 21; 9.50%), and so forth. The pA297-3 plasmid was found in all ST25 isolates and the RepAci4 plasmid was present in three ST126 isolates.

Besides plasmid identification, we also investigated insertion sequences (ISs) and integrons. For the ISs, we found that IS*Aba22* (n = 154; 69.68%) and IS*Ec29* (n = 153; 69.23%) were the most common ISs in the CRAB isolates. ISA*ba26*, IS*Vsa3*, IS*Aba1*, and ISA*ba24* were detected in 141 (63.80%), 138 (62.44%), 119 (53.85%), and 114 (51.58%) isolates, respectively. IS*Aba11* was only detected in all ST25 isolates, while other ISs were distributed in several ST isolates. The IS results are illustrated in Fig. 4 and S1, Table S6.

For the integrons, the integron-associated *intI1* gene was detected in 34 isolates (15.38%). The results also showed the arrangement of genes on integrons, particularly AMR genes. In 34 integron-positive isolates, 21 (61.76%), 2 (5.88%), and 1 (2.94%) isolates carried the *aac(6′)-Ib*, *aac(3)-Ia*, and *bla*_IMP-14_ genes on their integrons, respectively. Twenty (58.82%) isolates harbored an efflux pump gene on their integrons, which may provide chloramphenicol resistance, as shown in Fig. 4 and S1, Table S7.

### 3.5 Virulence-associated genes

**Fig. 5:**
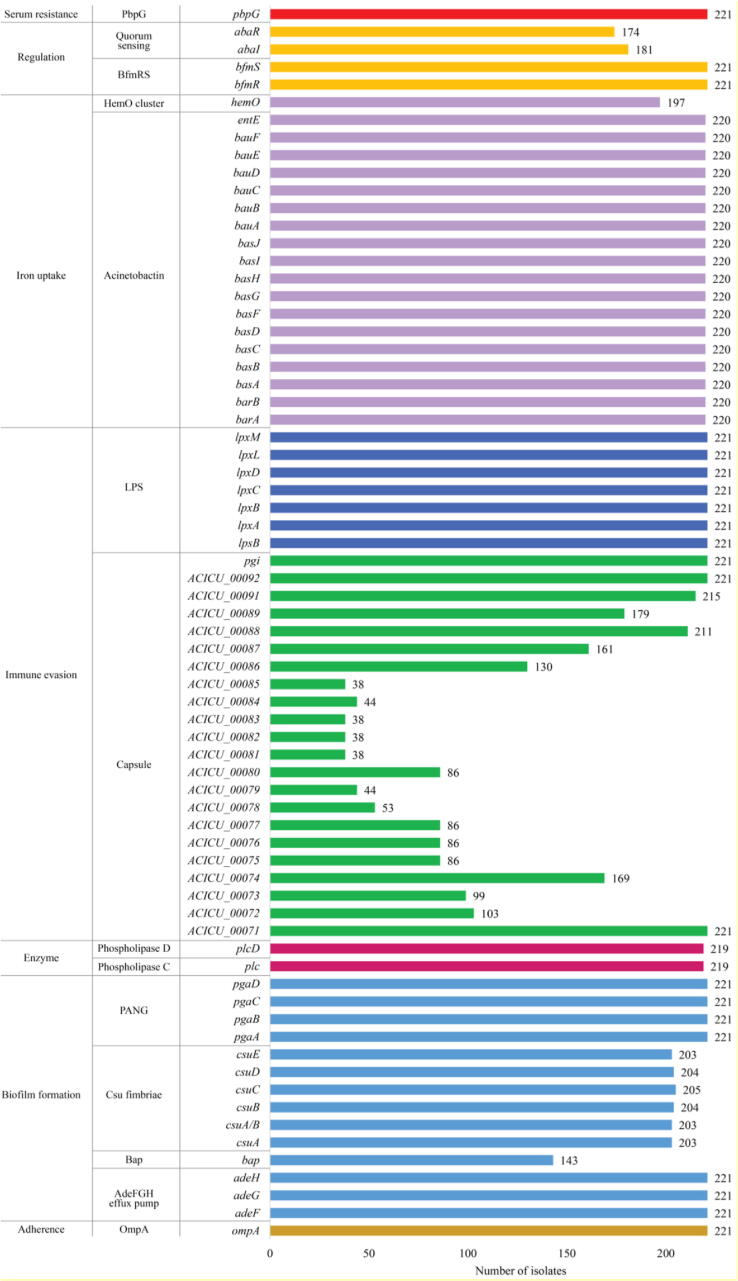
The presence of virulence-associated genes in the study 221 CRAB clinical isolates. OmpA, outer membrane protein A; Bap, biofilm-associated protein; PNAG, poly-N-acetylglucosamine; LPS, lipopolysaccharide; Bfm, biofilm formation; PbpG, penicillin-binding protein; *ACICU_0071* to *ACICU_0092* represent the genes encoding proteins related to the capsule (*ACICU_0071*, ATPase; *ACICU_0072*, protein-tyrosine-phosphatase; *ACICU_0073*, periplasmic protein; *ACICU_0074*, UDP-N-acetyl-D-mannosaminuronate dehydrogenase; *ACICU_0075*, nucleoside-diphosphate sugar epimerase; *ACICU_0076*, pyridoxal phosphate-dependent enzyme; *ACICU_0077*, CMP-N-acetylneuraminic acid synthetase; *ACICU_0078*, spore coat polysaccharide biosynthesis protein [glycosyltransferase]; *ACICU_0079*, acetyltransferase; *ACICU_0080*, sialic acid synthase; *ACICU_0081*, membrane protein; *ACICU_0082* - *ACICU_0085*, hypothetical protein; *ACICU_0086*, glycosyltransferase; *ACICU_0087*, sugar transferase; *ACICU_0088*, UDP-glucose pyrophosphorylase; *ACICU_0089*, UDP-glucose 6-dehydrogenase; *ACICU_0091*, UDP-glucose 4-epimerase; *ACICU_0092*, phosphomannomutase).

The investigation of the virulence factors of *Acinetobacter* spp. in the CRAB isolates revealed the presence of virulence genes associated with adherence, biofilm formation, enzyme, immune evasion, iron uptake, regulation, and serum resistance. All isolates possessed genes encoding OmpA (outer membrane protein A), AdeFGH efflux pump, PANG (poly-N-acetylglucosamine), LPS (lipopolysaccharide), BfmRS (regulation of biofilm formation), and PbpG (penicillin-binding protein). A total of 220/221 (99.55%) isolates (except SK066) harbored all acinetobactin genes. Twenty-six to 29 virulence genes were identified in 220 of the isolates, while only 10 virulence genes were detected in SK066. Among the capsule-encoding genes that are responsible for immune evasion, all isolates carried *ACICU_0071* (ATPase gene), *ACICU_0092* (phosphomannomutase gene), and *pgi* genes. *ACICU_0091* (UDP-glucose 4-epimerase gene), *ACICU_0088* (UDP-glucose pyrophosphorylase gene), *ACICU_0089* (UDP-glucose 6-dehydrogenase gene), *ACICU_0074* (UDP-N-acetyl-D-mannosaminuronate dehydrogenase gene), and *ACICU_0087* (sugar transferase gene) were mostly found in 215 (97.28%), 211 (95.48%), 179 (81.00%), 169 (76.47%), and 161 (72.85%) isolates. Likewise, the study showed that 219 (99.10%), 203 (91.86%) to 205 (92.76%), 197 (89.14%), 181 (81.90%) to 174 (78.73%), and 143 (64.71%) isolates possessed virulence factors including phospholipase D/C, Csu fimbriae, hemO cluster, quorum sensing, and biofilm-associated proteins, respectively. Virulence-associated gene information and results are shown in Fig. 5 and S2, Table S8.

### 3.6 Bacteriocin-encoding gene and bacteriophage genomes

In this study, we also explored the presence of bacteriocin and bacteriophages. For bacteriocin identification, the study showed that all CRAB isolates carried the *zooA* gene encoding zoocin A. In addition, we found the genome matching three phage families, belonging to the order *Caudovirales* in the prophage investigation. The *Siphoviridae*, *Myoviridae*, and *Podoviridae* families were seen in 212 (95.93%), 63 (28.51%), and 37 (16.74%) isolates, respectively. The bacteriocin-encoding gene and bacteriophage genome information is given in Fig. S2, Table S9.

### 3.7 Pan-genome and phylogenetic analysis

**Fig. 6:**
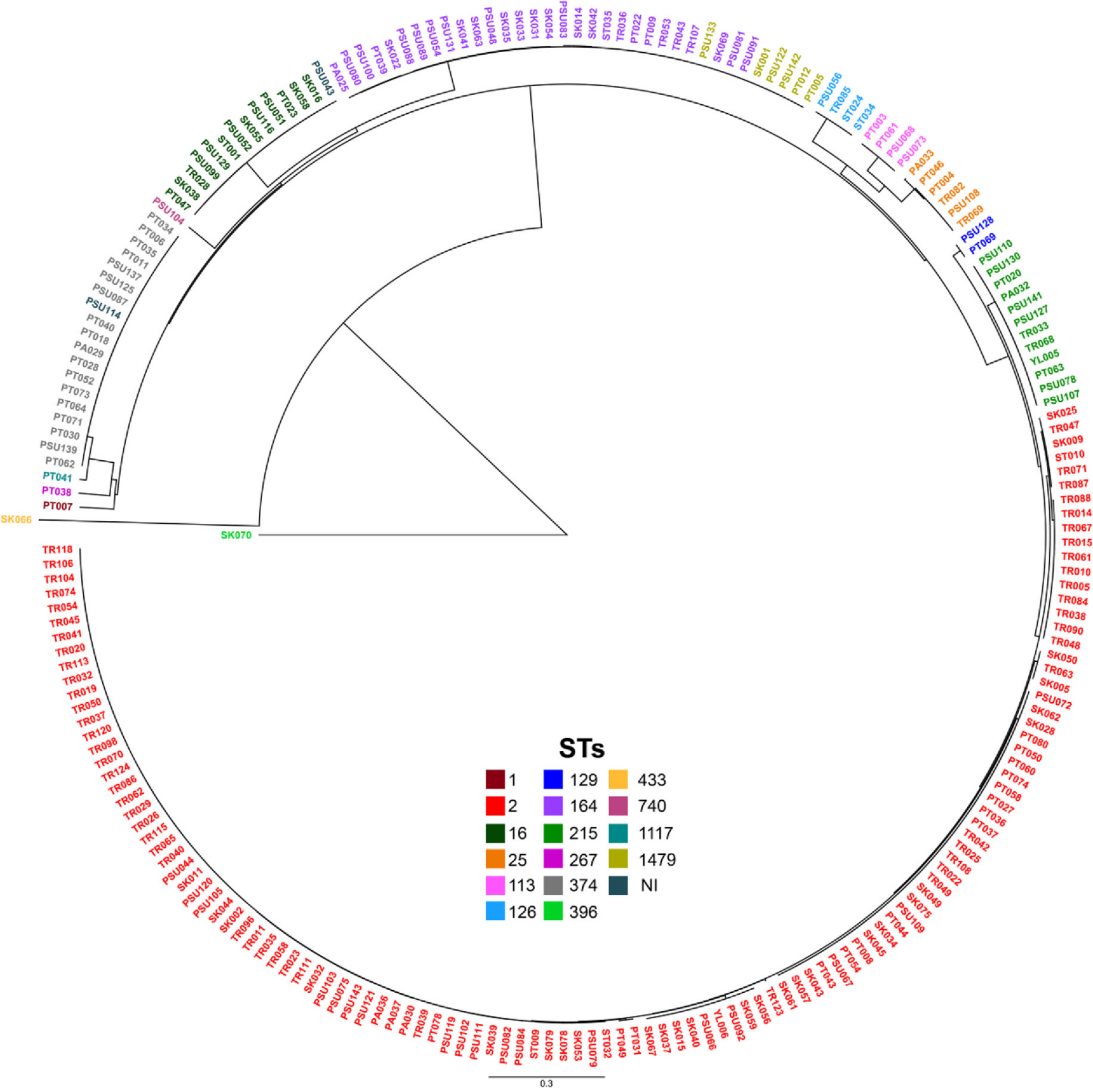
Phylogenetic tree constructed by calling SNPs from core gene alignment of the study 221 CRAB clinical isolates.

The results of pan-genome analysis among the study 221 CRAB isolates showed that 9,318 (68.68%), 2,412 (17.78%), 1,615 (11.90%), and 222 (1.64%) of 13,567 pan genes were identified as cloud, core, shell, and soft-core genes, respectively. These CRAB isolates contained various genes associated with transcriptional regulators and transporters in 3.96% and 4.19% of the pan-genome, respectively. Notably, we also found genes encoding transposases and bacteriophage proteins (e.g., heads, capsids, and tails) in 1.30% and 0.51% of the accessory genome. Furthermore, genes encoding hypothetical proteins were also observed in 5.97% and 66.44% of core and accessory genomes, respectively. Overall, the phylogenetic tree could be divided into many clades according to STs, as illustrated in Fig. 6 and S3. Among these clades, ST1479 was in the same clade as ST164. For two isolates that could not be assigned to an STs, one isolate was grouped into the ST374 clade, while the other isolate was located in the ST16 clade. Interestingly, we noticed that two isolates, SK066 and SK070, which were very different in terms of gene presence and absence, belonged to the different clades, as shown in the red box of Fig. S4. According to the gene presence and absence matrix (Fig. S4) and pan-genome graph (Fig. S5), the pan-genome profiles of these CRAB isolates seem to provide an open pan-genome with vast genomic diversity (Fig. S5).

In addition, the results of pan-genome analysis of our 221 CRAB genomes compared to 188 previously published genomes are exhibited in Figs. S6 - S9. In 18,915 pan genes, 14,685 (77.64%), 2,282 (12.06%), 1,633 (8.63%), and 315 (1.67%) genes were detected as cloud, core, shell, and soft-core genes, respectively. Genes encoding transposases and bacteriophage proteins (e.g., heads, capsids, and tails) were observed in 2.78% and 0.49% of the accessory genome, respectively.

## 4 Discussion

The rapid increase of CRAB infections seriously threatens the global population. Since most CRAB isolates resist many potential antimicrobial classes, it is currently difficult, if not impossible, to manage CRAB infections, especially through the use of suitable antibiotics. Faced with these challenges, many scientists across the world are trying to characterize and understand the mechanisms of antimicrobial resistance in CRAB isolates. We therefore used WGS as a comprehensive method for studying and exploring the genetic basis of 221 CRAB isolates collected at hospitals in Southern Thailand.

According to the results of this study, the largest proportion of CRAB isolates was identified in ST2, which belonged to international clone 2 (IC2) [53], [54]. ST2 is the most predominant type and the most widespread in many parts of the world, especially Thailand [55], [56], [57]. The prevalences of other STs such as ST164, ST374, ST16, ST215, ST25 (IC7), ST129 (IC2), and ST1 (IC1) are different in each region [55], [56], [58], [59], [60]. Hamidian and Nigro (2019) reported the level ST of 3575 CRAB isolates showing ST2 as the most prevalent ST, followed most prominently by ST1 (IC1), ST76, SLV2 (a single-locus variant of ST2), ST25 (IC7), ST10, and then others with lower prevalences [56]. Here, we also noticed that 6 isolates belonged to ST1479, a new ST that was recently discovered in a clinical isolate of extensively drug-resistant *A. baumannii* (XDRAB) from Thailand [61]. Unfortunately, two isolates could not be assigned to an ST by PubMLST in this study. PSU043 contained one nucleotide substitution (A99G) in the *recA* gene, which is a silent mutation that does not cause an alteration of the amino acid. Therefore, we predicted that this mutation may not change the function of the RecA protein, and the nearest ST identified by the PubMLST was ST16. For PSU114, although there were no mutations in all seven housekeeping genes, we still could not identify the ST.

In the detection of AMR genes, a majority of the CRAB isolates possessed the *bla*_OXA-23_ gene, which is the distinctive class D carbapenemase-encoding gene in *A. baumannii*. It has been reported in many countries all over the world including the USA, Australia, Germany, Brazil, China, Korea, Thailand, Vietnam, Malaysia, Pakistan, and Egypt [1], [3], [15], [16], [17], [18], [55], [56], [62], [63]. Normally, oxacillinase (OXA) enzymes have a weak hydrolyzing activity and are poorly expressed, resulting in a low level of carbapenem resistance [56], [64]. Nevertheless, the *bla*_OXA_ expression can be enhanced by an IS located upstream of the *bla*_OXA_ genes, leading to a high level of carbapenem resistance [56], [65]. As to the results of our study, IS*Aba1* was identified in 56.04% of the *bla*_OXA-23_-positive CRAB isolates. Many earlier reports have demonstrated that IS*Aba1* is generally located upstream of the *bla*_OXA-23-like_ gene, and it provides a strong promoter that drives the expression of the *bla*_OXA-23-like_ gene [63], [66], [67], [68]. In addition, we found other *bla*_OXA_ variants such as the *bla*_OXA-58_ gene. The OXA-58 enzyme has been reported with high-level resistance to carbapenem in *A. baumannii* [69]. More than half of our CRAB isolates also carried the *bla*_OXA-66_ gene, and some of the isolates harbored the *bla*_OXA-91_, *bla*_OXA-259_, and *bla*_OXA-402_ genes, and so forth. These genes are member of the *bla*_OXA-51-like_ genes, the intrinsic oxacillinase genes with low-level carbapenemase activity that naturally occur and are located on the chromosome of *A. baumannii* [62], [65], [70], [71].

In addition, some of our CRAB isolates possessed class B carbapenemase (metallo-β-lactamase; MBL) genes including *bla*_NDM-1_ and *bla*_IMP-14_ genes. These MBL genes provide a broad spectrum of carbapenemase activity in Gram-negative bacteria, particularly Enterobacterales, *Acinetobacter* spp., and *Pseudomonas aeruginosa* [72]. Additionally, we found IS*Aba125* in 72.22% of the *bla*_NDM-1_-positive CRAB isolates. IS*Aba125* is commonly located upstream and provides a promoter sequence for the *bla*_NDM-1_ expression [73], [74]. We also found extended-spectrum β-lactamase (ESBL) genes (*bla*_TEM-12_, *bla*_VEB-1_, *bla*_VEB-7_, *bla*_PER-1_, and *bla*_PER-7_) and other β-lactamase genes (e.g., *bla*_AmpC_, *bla*_ADC-6_, *bla*_ADC-30_, *bla*_ADC-73_, *bla*_ADC-79_, *bla*_CARB-16_, *ble*, etc.). The *bla*_AmpC_ and *bla*_ADC_ genes are the class C β-lactamase genes, while the *bla*_CARB_ gene is a class A β-lactamase gene. Overproduction of AmpC β-lactamase in combination with ESBLs, efflux pumps, and/or porin loss has been associated with carbapenem resistance in Gram-negative bacteria [75], [76].

Besides β-lactam resistance genes, the other genes that probably confer resistance to aminoglycoside, fosfomycin, tetracycline, chloramphenicol, trimethoprim, sulfonamide, lincosamide, and macrolide were found in these CRAB isolates and they might be classified as multidrug-resistant (MDR) isolates accordingly. Many studies have reported on the retained susceptibility to aminoglycosides (e.g., amikacin and gentamicin) and tetracyclines (e.g., doxycycline and tigecycline) in carbapenem-resistant Gram-negative bacteria (CR-GNB) [74], [77], [78], [79]. Unfortunately, although aminoglycosides and tetracyclines are used as a monotherapy, or in combination with other antimicrobial agents against CR-GNB infection [77], [80], aminoglycosides seem more effective in carbapenem-resistant Enterobacteriaceae (CRE) than in CRAB, while tetracyclines are normally used to combat both CRE and CRAB [80]. The tigecycline resistance is continuously found in CRAB [78]. Notably, although low-level resistance to plazomicin, a novel aminoglycoside, has been reported in CRAB [80], we found that over a haft of the CRAB isolates carried the *armA* gene, which has been reported as confer high-level resistance to aminoglycosides, particularly gentamicin, amikacin, tobramycin, and plazomicin [81]. Our CRAB isolates also harbored other aminoglycoside resistance genes (*aph(6)-Id*, *aph(3′)-Ia*, *aadA1*, *aac(3)-IId*, *ant(2″)-Ia*, *aac(6′)-lb*, *armA*, etc.) and tetracycline resistance genes (*tet*(B) and *tet*(39)), a finding concordant with previous studies [82], [83]. As well, previous studies demonstrated good efficacy of fosfomycin in combination with other antibiotics against CR-GNB [74], [84], [85]. However, our findings showed that the *abaF* gene, a fosfomycin resistance gene, was seen in almost all CRAB isolates. This would indicate that aminoglycosides, tetracyclines, and fosfomycin should be used with caution for the treatment of CRAB infections. In addition, the *mphE* and *msrE* genes were present in a high number of the CRAB isolates. These genes commonly associated with MDR in *A. baumannii*, which confer resistance to macrolide by inactivation (*mphE*) and modification of the target site (*msrE*) [86], [87].

In our study on the dissemination mechanisms of the AMR gene among Gram-negative bacteria, we investigated the MGEs (plasmids, IS elements, and integrons). The findings showed that the largest number of CRAB isolates harbored the RepAci1 and RepAci7 plasmids. The RepAci1 plasmid is one of the most widespread plasmids in *Acinetobacter* spp. [88]. Towner et al. (2011) reported that the *bla*_OXA-23-like_ and *bla*_OXA-58-like_ genes were associated with the carriage of the *repAci1* replicase gene located on the RepAci1 plasmid [71]. Surprisingly, here, we found a high prevalence of RepAci7 plasmid in these CRAB isolates, which has not been reported in any other countries. Loraine et al. (2020) previously analyzed the genomics of *A. baumannii* isolated from three hospitals located in Central and Southern Thailand. They found the RepAci1, RepAci6, and RepApAB49 plasmids to have a high frequencies, while the RepAci7 plasmid was not detected in any *A. baumannii* isolates. Additionally, plasmid pA297-3 was present in all ST25 isolates. This plasmid has been associated with the AMR spread in *Acinetobacter* spp., particularly in ST25 isolates [89]. Among the 61 ISs detected in our study, we found that IS*Aba22* was the most prevalent IS, followed by IS*Ec29*, IS*Aba26*, IS*Vsa3*, IS*Aba1*, and IS*Aba24*. Besides functioning as the promoters for the expression of many AMR genes, the ISs generally provide for a cut-and-paste mechanism of transposition [90]. Previous studies have shown that the *bla*_OXA-23_ gene is mostly located in a composite transposon Tn*2006* that is bracketed by two copies of IS*Aba1* [88], [90]. In terms of integrons, we found an integron-integrase gene (*IntI1*) in 34 CRAB isolates. The AMR genes, especially aminoglycoside resistance genes (*aac(6′)-Ib* and *aac(3)-Ia*), were present in the integron-encoded IntI1 integrase. The SK066 isolate carried the *bla*_IMP-14_ gene in the class 1 integron, similar to many other reports [91], [92]. According to the MGE results, we indicate that if the AMR genes locate on the MGEs, these particular genes might be horizontally transferred (conjugation) to other *Acinetobacter* spp. as well as other species of Gram-negative pathogenic bacteria. Thus, the MGEs are a significant factor in the acquisition and spread of AMR genes [93].

In addition to AMR, pathogenic bacteria have evolved and developed virulence to host-defense mechanisms [94], [95]. We found the virulence-associated genes encoding for many virulence factors (e.g., adherence, biofilm formation, enzyme, immune evasion, iron uptake, regulation, and serum resistance) in all CRAB isolates. This finding could imply that the presence of these virulence genes may increase the pathogenicity of these CRAB isolates and the severity of infection [95]. Importantly, they might be spread to other bacteria through horizontal gene transfer, similar to the AMR genes [94]. Furthermore, we found the *zooA* gene encoding zoocin A in all isolates. Zoocin A, a bacteriocin-like inhibitory substance (BLIS), was first identified in *Streptococcus equi* subsp. *zooepidemicus* strain 4881 [96], [97], [98]. It is a peptidoglycan hydrolase that is responsible for inhibiting the peptidoglycan synthesis in many other streptococcal species, especially *S. mutans*, *S. sobrinus*, and *S. cricetus* [96], [97], [98]. Importantly, it is also classified as a penicillin-binding protein (PBP) [96], which provides weak β-lactamase activity against penicillin. This might be one of the factors causing high-level β-lactam resistance in the CRAB isolates. In the investigation of the bacteriophage genome, a very high level of CRAB isolates showed sequence alignment to *Siphoviridae* phage, while some isolates also harbored *Myoviridae* and/or *Podoviridae* phages. These three phage families belong to the order *Caudovirales*, which contains double-stranded DNA (dsDNA) genomes [99]. We thus postulate that the CRAB isolates might have been previously infected by these particular phages. More importantly, the DNA phages can drive the bacterial genes, especially AMR genes, to other bacteria by generalized transduction [100], [101]. Thus, the discovery of these bacteriophage genomes within the CRAB genomes may be indicated as one of the factors causing the spread of AMR genes or other genes.

To demonstrate the genomic diversity, we analyzed the pan-genome among our 221 CRAB isolates. The pan-genome profiles showed that these CRAB isolates shared 17.78% core genes, and contained a very high percentage (68.68%) of cloud genes. In a presence and absence matrix of pan-genome, the accessory genome demonstrated the presence of genes encoding transposases and bacteriophage proteins (e.g., head, capsid, and tails), and the high level of genes encoding hypothetical proteins in these CRAB isolates. We hypothesized that these genes are probably involved in adaptation mechanisms, particularly the acquisition of AMR and virulence genes and the ability to persist in some changing environments [102], [103]. Based on our analysis, the pan-genome graph could be possibly considered as an open pan-genome since our dataset contained only 221 isolates. However, in the analysis of a larger dataset of *A. baumannii* isolates, the result also exhibited that exponential pan-genome growth was observed when increasing a great number of pan-genome [103], [104], [105], [106]. Furthermore, the high proportion of accessory genes demonstrated a high genomic diversity among these isolates. This discovery could be an indicator of a useful path for researchers to explore and perhaps find new genes and to study genomic diversity in the *A. baumannii* strains, especially carbapenem-resistant and MDR isolates. Notably, two isolates (SK066 and SK070) in our study were uncommon and very different from the other CRAB isolates. However, after submitting the genome sequences of 221 isolates into the NCBI server, the submission details reported that only 219 isolates were CRAB, whereas the other 2 isolates belonged to other *Acinetobacter* species. The average nucleotide identity (ANI) results from NCBI revealed that the SK066 and SK077 isolates were identified as *Acinetobacter pittii* (97.42% identity) and *Acinetobacter nosocomialis* (97.85% identity), respectively. These two isolates were previously confirmed as *A. baumannii* by both *in vitro* and *in silico* methods and also carried carbapenem resistance genes as well as other AMR genes. This could indicate that these genomes are biologically related among *Acinetobacter* spp. To further elucidate the genomic data of these two isolates, additional tools and technologies such as long-read WGS should be considered to provide more information in the future. In addition, the pan-genome analysis of our CRAB genomes compared to previously published genomes demonstrated that the specific features could not be observed among the accessory genomes. These findings could be indicated that genetic features of the *A. baumannii* clinical isolates from Thailand are closely related.

WGS provides more accurate details and more precise information than traditional microbiological methods; for example, in the comparison of antimicrobial susceptibility patterns obtained from antimicrobial susceptibility testing (AST) and bacterial DNA fingerprints obtained from pulse-field gel electrophoresis (PFGE) [107]. These strengths of this method allow scientists to compare entire genome sequences within the bacterial cell, for a better understanding of the epidemiology of the pathogens such as identifying the environmental source of an outbreak, transmission events, and the mechanism for spreading antimicrobial resistance [108], [109], [110]. Since the presence or absence of MGEs carrying AMR genes results in genetic variation and changes in antimicrobial susceptibility patterns, WGS can rapidly provide crucial data during an acute outbreak [111]. Therefore, using WGS to study the genomic insights into the pathogens, especially antibiotic-resistant strains, could be beneficial for outbreak investigations and surveillance as well as infection control and prevention.

## 5 Conclusions

This study revealed significant information from a short-read WGS analysis. All CRAB isolates possessed various AMR genes, MGEs (especially plasmids and ISs), and virulence-associated genes, which can be horizontally transferred to other pathogenic bacteria causing widespread carbapenem resistance. The bacteriocin gene and the bacteriophage genomes were present at the highest frequency in these CRAB isolates. Finally, a way to rapidly identify and characterize the genomic features of the CR-GNB strains is necessary before we will be able to finally control the spread of these pathogens in the future.

## 6 Data availability statement

The assembled genomes of all 221 CRAB isolates have been deposited in the NCBI GenBank under BioProject number PRJNA752484 with BioSample numbers SAMN20599216 to SAMN20599436.

## Funding

This research was funded by the National Science and Technology Development Agency (NSTDA), Thailand (project number: P-20–51325) and the Graduate Scholarship, Faculty of Medicine, Prince of Songkla University, Thailand (grant number: 62-012). This study was also supported by the Faculty of Science, Prince of Songkla University, Thailand (grant number: SCI64040135).

## Ethical approval

The study was approved by the Human Research Ethics Committee (HREC) of Prince of Songkla University (reference number: 64–284-14–1, date of approval: 9 June 2021).

## CRediT authorship contribution statement

**Arnon Chukamnerd:** Conceptualization, Methodology, Software, Validation, Formal analysis, Investigation, Resources, Data curation, Writing – original draft, Visualization, Project administration. **Kamonnut Singkhamanan:** Methodology, Resources, Writing – review & editing, Supervision, Funding acquisition. **Virasakdi Chongsuvivatwong:** Conceptualization, Data curation, Writing – review & editing, Supervision, Funding acquisition. **Prasit Palittapongarnpim:** Data curation, Writing – review & editing, Supervision. **Yohei Doi:** Writing – review & editing, Supervision. **Rattanaruji Pomwised:** Methodology, Resources, Writing – review & editing, Supervision. **Chanida Sakunrang:** Methodology, Validation, Investigation, Resources. **Kongpop Jeenkeawpiam:** Methodology, Software, Investigation, Resources. **Mingkwan Yingkajorn:** Resources, Funding acquisition. **Sarunyou Chusri:** Conceptualization, Methodology, Formal analysis, Investigation, Resources, Data curation, Writing – original draft, Writing – review & editing, Visualization, Supervision, Project administration, Funding acquisition. **Komwit Surachat:** Conceptualization, Methodology, Software, Validation, Formal analysis, Investigation, Resources, Data curation, Writing – original draft, Writing – review & editing, Visualization, Supervision, Project administration, Funding acquisition.

## Declaration of Competing Interest

The authors declare that they have no known competing financial interests or personal relationships that could have appeared to influence the work reported in this paper.

## References

[1] Kostyanev T., Xavier B.B., García-Castillo M., Lammens C., Acosta J.B.-F. (2021). Phenotypic and molecular characterizations of carbapenem-resistant Acinetobacter baumannii isolates collected within the EURECA study. Int J Antimicrob Agents.

[2] Pogue J.M., Mann T., Barber K.E., Kaye K.S. (2013). Carbapenem-resistant *Acinetobacter baumannii*: epidemiology, surveillance and management. Expert Rev Anti Infect Ther.

[3] Wareth G., Linde J., Nguyen N.H., Nguyen T.N., Sprague L.D. (2021). WGS-based analysis of carbapenem-resistant *Acinetobacter baumannii* in Vietnam and molecular characterization of antimicrobial determinants and MLST in Southeast Asia. Antibiotics.

[4] Pormohammad A., Mehdinejadiani K., Gholizadeh P., Nasiri M.J., Mohtavinejad N. (2020). Global prevalence of colistin resistance in clinical isolates of *Acinetobacter baumannii*: A systematic review and meta-analysis. Microb Pathog.

[5] Theriault N., Tillotson G., Sandrock C.E. (2021). Global travel and Gram-negative bacterial resistance; implications on clinical management. Expert Rev Anti Infect Ther.

[6] Centers for Disease Control and Prevention (CDC). Antibiotic Resistance Threats in the United States, 2019 (2019 AR Threats Report), CDC, Atlanta, GA. 2019. [Accessed 04 January 2020]; Available from: https://www.cdc.gov/drugresistance/Biggest-Threats.html.

[7] Ambrosi C., Scribano D., Aleandri M., Zagaglia C., Di Francesco L. (2017). *Acinetobacter baumannii* virulence traits: a comparative study of a novel sequence type with other Italian endemic international clones. Front Microbiol.

[8] Cerqueira G.M., Peleg A.Y. (2011). Insights into *Acinetobacter baumannii* pathogenicity. IUBMB Life.

[9] Ambler R. (1980). The structure of β-lactamases. Phil Trans R Soc Lond B.

[10] Voulgari E., Zarkotou O., Ranellou K., Karageorgopoulos D.E., Vrioni G. (2012). Outbreak of OXA-48 carbapenemase-producing *Klebsiella pneumoniae* in Greece involving an ST11 clone. J Antimicrob Chemother.

[11] Jiang L., Yu Y., Zeng W., Guo J., Lv F. (2020). Whole-genome analysis of New Delhi Metallo-Beta-Lactamase-1-producing *Acinetobacter haemolyticus* from China. J Glob Antimicrob Resist.

[12] Liu B.-T., Su W.-Q. (2019). Whole genome sequencing of NDM-1-producing serotype K1 ST23 hypervirulent *Klebsiella pneumoniae* in China. J Med Microbiol.

[13] Mansour W., Grami R., Jaidane N., Messaoudi A., Charfi K. (2019). Epidemiology and whole-genome analysis of NDM-1-producing *Klebsiella pneumoniae* KP3771 from Tunisia. Microb Drug Resist.

[14] Runcharoen C., Raven K.E., Reuter S., Kallonen T., Paksanont S. (2017). Whole genome sequencing of ESBL-producing *Escherichia coli* isolated from patients, farm waste and canals in Thailand. Genome Med.

[15] Kim M.H., Jeong H., Sim Y.M., Lee S., Yong D. (2020). Using comparative genomics to understand molecular features of carbapenem-resistant *Acinetobacter baumannii* from South Korea causing invasive infections and their clinical implications. PLoS ONE.

[16] Loraine J., Heinz E., Soontarach R., Blackwell G.A., Stabler R.A. (2020). Genomic and phenotypic analyses of *Acinetobacter baumannii* isolates from three tertiary care hospitals in Thailand. Front Microbiol.

[17] Rao M., Rashid F.A., Shukor S., Hashim R., Ahmad N. (2020). Detection of antimicrobial resistance genes associated with carbapenem resistance from the whole-genome sequence of *Acinetobacter baumannii* isolates from Malaysia. Can J Infect Dis Med Microbiol.

[18] Camargo C.H., Cunha M.P.V., de Barcellos T.A.F., Bueno M.S., de Jesus Bertani A.M. (2020). Genomic and phenotypic characterisation of antimicrobial resistance in carbapenem-resistant *Acinetobacter baumannii* hyperendemic clones CC1, CC15, CC79 and CC25. Int J Antimicrob Agents.

[19] Krieg N.R., Holt J.G. (1984).

[20] Espinal P., Seifert H., Dijkshoorn L., Vila J., Roca I. (2012). Rapid and accurate identification of genomic species from the *Acinetobacter baumannii* (Ab) group by MALDI-TOF MS. Clin Microbiol Infect.

[21] MarÝ-Almirall M, Cosgaya C, Higgins PG, Van Assche A, Telli M, et al. MALDI-TOF/MS identification of species from the *Acinetobacter baumannii* (Ab) group revisited: inclusion of the novel *A. áseifertii* and *A. ádijkshoorniae* species. Clin Microbiol Infect 2017;23(3):210.e1-210.e9. 10.1016/j.cmi.2016.11.020 27919649

[22] Clinical and Laboratory Standards Institute (CLSI). Performance standards for antimicrobial susceptibility testing, 28th ed; approved standard M100. CLSI, Wayne, PA. 2018.

[23] Bankevich A., Nurk S., Antipov D., Gurevich A.A., Dvorkin M. (2012). SPAdes: a new genome assembly algorithm and its applications to single-cell sequencing. J Comput Biol.

[24] Gurevich A., Saveliev V., Vyahhi N., Tesler G. (2013). QUAST: quality assessment tool for genome assemblies. Bioinformatics.

[25] Simão F.A., Waterhouse R.M., Ioannidis P., Kriventseva E.V., Zdobnov E.M. (2015). BUSCO: assessing genome assembly and annotation completeness with single-copy orthologs. Bioinformatics.

[26] Seppey M., Manni M., Zdobnov E.M. (2019). BUSCO: assessing genome assembly and annotation completeness. Methods Mol Biol (Clifton, NJ).

[27] Seemann T. (2014). Prokka: rapid prokaryotic genome annotation. Bioinformatics.

[28] Chan PP, Lowe TM, tRNAscan-SE: searching for tRNA genes in genomic sequences. In Gene prediction (pp. 1-14). Humana, New York, NY. 10.1007/978-1-4939-9173-0_1 PMC6768409 31020551

[29] Lowe T.M., Chan P.P. (2016). tRNAscan-SE On-line: integrating search and context for analysis of transfer RNA genes. Nucleic Acids Res.

[30] Lagesen K., Hallin P., Rødland E.A., Stærfeldt H.-H., Rognes T. (2007). RNAmmer: consistent and rapid annotation of ribosomal RNA genes. Nucleic Acids Res.

[31] Larsen M.V., Cosentino S., Lukjancenko O., Saputra D., Rasmussen S. (2014). Benchmarking of methods for genomic taxonomy. J Clin Microbiol.

[32] Jolley KA, Bray JE, Maiden MC. Open-access bacterial population genomics: BIGSdb software, the PubMLST. org website and their applications. Wellcome Open Res 2018;3:124. 10.12688/wellcomeopenres.14826.1 PMC6192448 30345391

[33] Alcock B.P., Raphenya A.R., Lau T.T., Tsang K.K., Bouchard M. (2020). CARD 2020: antibiotic resistome surveillance with the comprehensive antibiotic resistance database. Nucleic Acids Res.

[34] Bertini A., Poirel L., Mugnier P.D., Villa L., Nordmann P. (2010). Characterization and PCR-based replicon typing of resistance plasmids in *Acinetobacter baumannii*. Antimicrob Agents Chemother.

[35] Salto I.P., Tejerizo G.T., Wibberg D., Pühler A., Schlüter A. (2018). Comparative genomic analysis of *Acinetobacter* spp. plasmids originating from clinical settings and environmental habitats. Sci Rep.

[36] Gao F., Wang Y., Liu Y.-J., Wu X.-M., Lv X. (2011). Genome sequence of *Acinetobacter baumannii* MDR-TJ. J Bacteriol.

[37] Hamidian M., Nigro S.J., Hall R.M. (2012). Variants of the gentamicin and tobramycin resistance plasmid pRAY are widely distributed in *Acinetobacter*. J Antimicrob Chemother.

[38] Hamidian M., Ambrose S.J., Hall R.M. (2016). A large conjugative *Acinetobacter baumannii* plasmid carrying the *sul2* sulphonamide and *strAB* streptomycin resistance genes. Plasmid.

[39] Zhang W.-J., Lu Z., Schwarz S., Zhang R.-M., Wang X.-M. (2013). Complete sequence of the *bla*_NDM-1_-carrying plasmid pNDM-AB from *Acinetobacter baumannii* of food animal origin. J Antimicrob Chemother.

[40] Jones L.S., Toleman M.A., Weeks J.L., Howe R.A., Walsh T.R. (2014). Plasmid carriage of *bla*_NDM-1_ in clinical *Acinetobacter baumannii* isolates from India. Antimicrob Agents Chemother.

[41] Blackwell G.A., Hall R.M. (2017). The *tet39* determinant and the *msrE-mphE* genes in *Acinetobacter* plasmids are each part of discrete modules flanked by inversely oriented p dif (XerC-XerD) sites. Antimicrob Agents Chemother.

[42] Hamidian M., Nigro S.J., Hartstein R.M., Hall R.M. (2017). RCH51, a multiply antibiotic-resistant *Acinetobacter baumannii* ST103IP isolate, carries resistance genes in three plasmids, including a novel potentially conjugative plasmid carrying *oxa235* in transposon Tn *6252*. J Antimicrob Chemother.

[43] Siguier P., Pérochon J., Lestrade L., Mahillon J., Chandler M. (2006). ISfinder: the reference centre for bacterial insertion sequences. Nucleic Acids Res.

[44] Cury J., Jové T., Touchon M., Néron B., Rocha E.P. (2016). Identification and analysis of integrons and cassette arrays in bacterial genomes. Nucleic Acids Res.

[45] Chen L., Yang J., Yu J., Yao Z., Sun L. (2005). VFDB: a reference database for bacterial virulence factors. Nucleic Acids Res.

[46] de Jong A., van Hijum S.A., Bijlsma J.J., Kok J., Kuipers O.P. (2006). BAGEL: a web-based bacteriocin genome mining tool. Nucleic Acids Res.

[47] Starikova E.V., Tikhonova P.O., Prianichnikov N.A., Rands C.M., Zdobnov E.M. (2020). Phigaro: high-throughput prophage sequence annotation. Bioinformatics.

[48] Page A.J., Cummins C.A., Hunt M., Wong V.K., Reuter S. (2015). Roary: rapid large-scale prokaryote pan genome analysis. Bioinformatics.

[49] Page A.J., Taylor B., Delaney A.J., Soares J., Seemann T. (2016). SNP-sites: rapid efficient extraction of SNPs from multi-FASTA alignments. Microb Genom.

[50] Stamatakis A. (2014). RAxML version 8: a tool for phylogenetic analysis and post-analysis of large phylogenies. Bioinformatics.

[51] Kearse M., Moir R., Wilson A., Stones-Havas S., Cheung M. (2012). Geneious Basic: an integrated and extendable desktop software platform for the organization and analysis of sequence data. Bioinformatics.

[52] Hadfield J., Croucher N.J., Goater R.J., Abudahab K., Aanensen D.M. (2018). Phandango: an interactive viewer for bacterial population genomics. Bioinformatics.

[53] Levy-Blitchtein S., Roca I., Plasencia-Rebata S., Vicente-Taboada W., Velásquez-Pomar J. (2018). Emergence and spread of carbapenem-resistant *Acinetobacter baumannii* international clones II and III in Lima, Peru. Emerg Microbes Infect.

[54] Giannouli M., Antunes L.C., Marchetti V., Triassi M., Visca P. (2013). Virulence-related traits of epidemic *Acinetobacter baumannii* strains belonging to the international clonal lineages I-III and to the emerging genotypes ST25 and ST78. BMC Infect Dis.

[55] Eigenbrod T., Reuter S., Gross A., Kocer K., Günther F. (2019). Molecular characterization of carbapenem-resistant *Acinetobacter baumannii* using WGS revealed missed transmission events in Germany from 2012–15. J Antimicrob Chemother.

[56] Hamidian M., Nigro S.J. (2019). Emergence, molecular mechanisms and global spread of carbapenem-resistant *Acinetobacter baumannii*. Microb Genom.

[57] Thirapanmethee K., Srisiri-A-Nun T., Houngsaitong J., Montakantikul P., Khuntayaporn P. (2020). Prevalence of OXA-Type β-Lactamase genes among carbapenem-resistant *Acinetobacter baumannii* clinical isolates in Thailand. Antibiotics.

[58] Cerezales M., Xanthopoulou K., Wille J., Bustamante Z., Seifert H. (2019). *Acinetobacter baumannii* analysis by core genome multi-locus sequence typing in two hospitals in Bolivia: endemicity of international clone 7 isolates (CC25). Int J Antimicrob Agents.

[59] Caldart R.V., Fonseca E.L., Freitas F., Rocha L., Vicente A.C. (2019). *Acinetobacter baumannii* infections in Amazon Region driven by extensively drug resistant international clones, 2016–2018. Mem Inst Oswaldo Cruz.

[60] Shelenkov A., Petrova L., Zamyatin M., Mikhaylova Y., Akimkin V. (2021). Diversity of international high-risk clones of *Acinetobacter baumannii* revealed in a Russian multidisciplinary medical center during 2017–2019. Antibiotics.

[61] Chopjitt P., Wongsurawat T., Jenjaroenpun P., Boueroy P., Hatrongjit R. (2020). Complete genome sequences of four extensively drug-resistant *Acinetobacter baumannii* isolates from Thailand. Microbiol Resour Announc.

[62] Abouelfetouh A., Torky A.S., Aboulmagd E. (2019). Phenotypic and genotypic characterization of carbapenem-resistant *Acinetobacter baumannii* isolates from Egypt. Antimicrob Resist Infect Control.

[63] Khurshid M., Rasool M.H., Ashfaq U.A., Aslam B., Waseem M. (2020). Dissemination of *bla*_OXA-23_-harbouring carbapenem-resistant *Acinetobacter baumannii* clones in Pakistan. J Glob Antimicrob Resist.

[64] Héritier C., Poirel L., Lambert T., Nordmann P. (2005). Contribution of acquired carbapenem-hydrolyzing oxacillinases to carbapenem resistance in *Acinetobacter baumannii*. Antimicrob Agents Chemother.

[65] Turton J.F., Ward M.E., Woodford N., Kaufmann M.E., Pike R. (2006). The role of IS*Aba1* in expression of OXA carbapenemase genes in *Acinetobacter baumannii*. FEMS Microbiol Lett.

[66] Khurshid M., Rasool M.H., Ashfaq U.A., Aslam B., Waseem M. (2017). Emergence of IS*Aba1* harboring carbapenem-resistant *Acinetobacter baumannii* isolates in Pakistan. Future Microbiol.

[67] Liu L.-L., Ji S.-J., Ruan Z., Fu Y., Fu Y.-Q. (2015). Dissemination of *bla*_OXA-23_ in *Acinetobacter* spp. in China: main roles of conjugative plasmid pAZJ221 and transposon Tn*2009*. Antimicrob Agents Chemother.

[68] Lin M.-F., Kuo H.-Y., Yeh H.-W., Yang C.-M., Sung C.-H. (2011). Emergence and dissemination of *bla*_OXA-23_-carrying imipenem-resistant *Acinetobacter* sp. in a regional hospital in Taiwan. J Microbiol Immunol Infect.

[69] Bertini A., Poirel L., Bernabeu S., Fortini D., Villa L. (2007). Multicopy *bla*_OXA-58_ gene as a source of high-level resistance to carbapenems in *Acinetobacter baumannii*. Antimicrob Agents Chemother.

[70] Héritier C., Poirel L., Fournier P.-E., Claverie J.-M., Raoult D. (2005). Characterization of the naturally occurring oxacillinase of *Acinetobacter baumannii*. Antimicrob Agents Chemother.

[71] Towner K.J., Evans B., Villa L., Levi K., Hamouda A. (2011). Distribution of intrinsic plasmid replicase genes and their association with carbapenem-hydrolyzing class D β-lactamase genes in European clinical isolates of *Acinetobacter baumannii*. Antimicrob Agents Chemother.

[72] Hammoudi Halat D., Ayoub M.C. (2020). The current burden of carbapenemases: review of significant properties and dissemination among gram-negative bacteria. Antibiotics.

[73] Xie L., Dou Y., Zhou K., Chen Y., Han L. (2017). Coexistence of *bla*_OXA-48_ and truncated *bla*_NDM-1_ on different plasmids in a *Klebsiella pneumoniae* isolate in China. Front Microbiol.

[74] Chukamnerd A., Pomwised R., Phoo M.T.P., Terbtothakun P., Hortiwakul T. (2021). *In vitro* synergistic activity of fosfomycin in combination with other antimicrobial agents against carbapenem-resistant *Klebsiella pneumoniae* isolated from patients in a hospital in Thailand. J Infect Chemother.

[75] Quale J., Bratu S., Landman D., Heddurshetti R. (2003). Molecular epidemiology and mechanisms of carbapenem resistance in *Acinetobacter baumannii* endemic in New York City. Clin Infect Dis.

[76] Mammeri H., Nordmann P., Berkani A., Eb F. (2008). Contribution of extended-spectrum AmpC (ESAC) β-lactamases to carbapenem resistance in *Escherichia coli*. FEMS Microbiol Lett.

[77] Qu J., Feng C., Li H., Lv X. (2021). Antibiotic strategies and clinical outcomes for patients with carbapenem-resistant Gram-negative bacterial bloodstream infection. Int J Antimicrob Agents.

[78] Piperaki E.-T., Tzouvelekis L., Miriagou V., Daikos G. (2019). Carbapenem-resistant *Acinetobacter baumannii*: in pursuit of an effective treatment. Clin Microbiol Infect.

[79] Hrenovic J., Seruga Music M., Durn G., Dekic S., Hunjak B. (2019). Carbapenem-resistant *Acinetobacter baumannii* recovered from swine manure. Microb Drug Resist.

[80] Bassetti M., Peghin M., Vena A., Giacobbe D.R. (2019). Treatment of infections due to MDR Gram-negative bacteria. Front Med.

[81] Nie L., Lv Y., Yuan M., Hu X., Nie T. (2014). Genetic basis of high level aminoglycoside resistance in *Acinetobacter baumannii* from Beijing, China. Acta Pharm Sin B.

[82] Selasi G.N., Nicholas A., Jeon H., Lee Y.C., Yoo J.R. (2015). Genetic basis of antimicrobial resistance and clonal dynamics of carbapenem-resistant *Acinetobacter baumannii* sequence type 191 in a Korean hospital. Infect, Genet Evol.

[83] Kuo S.-C., Huang W.-C., Huang T.-W., Wang H.-Y., Lai J.-F. (2018). Molecular epidemiology of emerging *bla*_OXA-23-like_- and *bla*_OXA-24-like_-carrying *Acinetobacter baumannii* in Taiwan. Antimicrob Agents Chemother.

[84] Pontikis K., Karaiskos I., Bastani S., Dimopoulos G., Kalogirou M. (2014). Outcomes of critically ill intensive care unit patients treated with fosfomycin for infections due to pandrug-resistant and extensively drug-resistant carbapenemase-producing Gram-negative bacteria. Int J Antimicrob Agents.

[85] Sirijatuphat R., Thamlikitkul V. (2014). Preliminary study of colistin versus colistin plus fosfomycin for treatment of carbapenem-resistant *Acinetobacter baumannii* infections. Antimicrob Agents Chemother.

[86] Kyriakidis I., Vasileiou E., Pana Z.D., Tragiannidis A. (2021). *Acinetobacter baumannii* Antibiotic Resistance Mechanisms. Pathogens.

[87] Cheng Y., Yang S., Jia M., Zhao L., Hou C. (2016). Comparative study between macrolide regulatory proteins MphR(A) and MphR(E) in ligand identification and DNA binding based on the rapid *in vitro* detection system. Anal Bioanal Chem.

[88] Blackwell G.A., Hall R.M. (2019). Mobilisation of a small *Acinetobacter* plasmid carrying an oriT transfer origin by conjugative RepAci6 plasmids. Plasmid.

[89] Nigro S.J., Hall R.M. (2017). A large plasmid, p D46–4, carrying a complex resistance region in an extensively antibiotic-resistant ST25 *Acinetobacter baumanni*i. J Antimicrob Chemother.

[90] Yoon E.-J., Kim J.O., Yang J.W., Kim H.S., Lee K.J. (2017). The *bla*_OXA-23_-associated transposons in the genome of *Acinetobacter* spp. represent an epidemiological situation of the species encountering carbapenems. J Antimicrob Chemother.

[91] Samuelsen Ø., Toleman M.A., Sundsfjord A., Rydberg J., Leegaard T.M. (2010). Molecular epidemiology of metallo-β-lactamase-producing *Pseudomonas aeruginosa* isolates from Norway and Sweden shows import of international clones and local clonal expansion. Antimicrob Agents Chemother.

[92] Stoesser N., Sheppard A.E., Peirano G., Sebra R.P., Lynch T. (2016). First report of *bla*_IMP-14_ on a plasmid harboring multiple drug resistance genes in *Escherichia coli* sequence type 131. Antimicrob Agents Chemother.

[93] Partridge S.R., Kwong S.M., Firth N., Jensen S.O. (2018). Mobile genetic elements associated with antimicrobial resistance. Clin Microbiol Rev.

[94] de Nies L., Lopes S., Busi S.B., Galata V., Heintz-Buschart A. (2021). PathoFact: a pipeline for the prediction of virulence factors and antimicrobial resistance genes in metagenomic data. Microbiome.

[95] Liu C., Chang Y., Xu Y., Luo Y., Wu L. (2018). Distribution of virulence-associated genes and antimicrobial susceptibility in clinical *Acinetobacter baumannii* isolates. Oncotarget.

[96] Heath L.S., Heath H.E., LeBlanc P.A., Smithberg S.R., Dufour M. (2004). The streptococcolytic enzyme zoocin A is a penicillin-binding protein. FEMS Microbiol Lett.

[97] Simmonds R.S., Simpson W.J., Tagg J.R. (1997). Cloning and sequence analysis of *zooA*, a Streptococcus zooepidemicus gene encoding a bacteriocin-like inhibitory substance having a domain structure similar to that of lysostaphin. Gene.

[98] Simmonds R., Naidoo J., Jones C., Tagg J. (1995). The streptococcal bacteriocin-like inhibitory substance, zoocin A, reduces the proportion of *Streptococcus* mutans in an artificial plaque. Microb Ecol Health Dis.

[99] Wintachai P., Naknaen A., Pomwised R., Voravuthikunchai S.P., Smith D.R. (2019). Isolation and characterization of Siphoviridae phage infecting extensively drug-resistant *Acinetobacter baumannii* and evaluation of therapeutic efficacy *in vitro* and *in vivo*. J Med Microbiol.

[100] Stanczak-Mrozek K.I., Laing K.G., Lindsay J.A. (2017). Resistance gene transfer: induction of transducing phage by sub-inhibitory concentrations of antimicrobials is not correlated to induction of lytic phage. J Antimicrob Chemother.

[101] Volkova V.V., Lu Z., Besser T., Gröhn Y.T. (2014). Modeling the infection dynamics of bacteriophages in enteric *Escherichia coli*: estimating the contribution of transduction to antimicrobial gene spread. Appl Environ Microbiol.

[102] Chan A.P., Sutton G., DePew J., Krishnakumar R., Choi Y. (2015). A novel method of consensus pan-chromosome assembly and large-scale comparative analysis reveal the highly flexible pan-genome of *Acinetobacter baumannii*. Genome Biol.

[103] Antunes L., Visca P., Towner K.J. (2014). *Acinetobacter baumannii*: evolution of a global pathogen. Pathog Dis.

[104] Mangas E.L., Rubio A., Álvarez-Marín R., Labrador-Herrera G., Pachón J. (2019). Pangenome of *Acinetobacter baumannii* uncovers two groups of genomes, one of them with genes involved in CRISPR/Cas defence systems associated with the absence of plasmids and exclusive genes for biofilm formation. Microb Genom.

[105] Rodrigues D.L.N., Morais-Rodrigues F., Hurtado R., Dos Santos R.G., Costa D.C. (2021). Pan-resistome insights into the multidrug resistance of *Acinetobacter baumannii*. Antibiotics.

[106] Hassan A., Naz A., Obaid A., Paracha R.Z., Naz K. (2016). Pangenome and immuno-proteomics analysis of *Acinetobacter baumannii* strains revealed the core peptide vaccine targets. BMC Genet.

[107] Kumar P., Sundermann A.J., Martin E.M., Snyder G.M., Marsh J.W. (2021). Method for economic evaluation of bacterial whole genome sequencing surveillance compared to standard of care in detecting hospital outbreaks. Clin Infect Dis.

[108] Quick J., Cumley N., Wearn C.M., Niebel M., Constantinidou C. (2014). Seeking the source of *Pseudomonas aeruginosa* infections in a recently opened hospital: an observational study using whole-genome sequencing. BMJ Open.

[109] Halachev M.R., Chan J.Z., Constantinidou C.I., Cumley N., Bradley C. (2014). Genomic epidemiology of a protracted hospital outbreak caused by multidrug-resistant *Acinetobacter baumannii* in Birmingham, England. Genome Med.

[110] Stoesser N., Sheppard A., Shakya M., Sthapit B., Thorson S. (2015). Dynamics of MDR *Enterobacter cloacae* outbreaks in a neonatal unit in Nepal: insights using wider sampling frames and next-generation sequencing. J Antimicrob Chemother.

[111] Quick J., Ashton P., Calus S., Chatt C., Gossain S. (2015). Rapid draft sequencing and real-time nanopore sequencing in a hospital outbreak of Salmonella. Genome Biol.

