# Supplementary figures and images for: Whole-genome analysis of carbapenem-resistant Acinetobacter baumannii from clinical isolates in Southern Thailand
Source: Comput Struct Biotechnol J. 2022 Jan 6;20:545–58. doi: 10.1016/j.csbj.2021.12.038 (PMC9582705; doi:10.1016/j.csbj.2021.12.038)

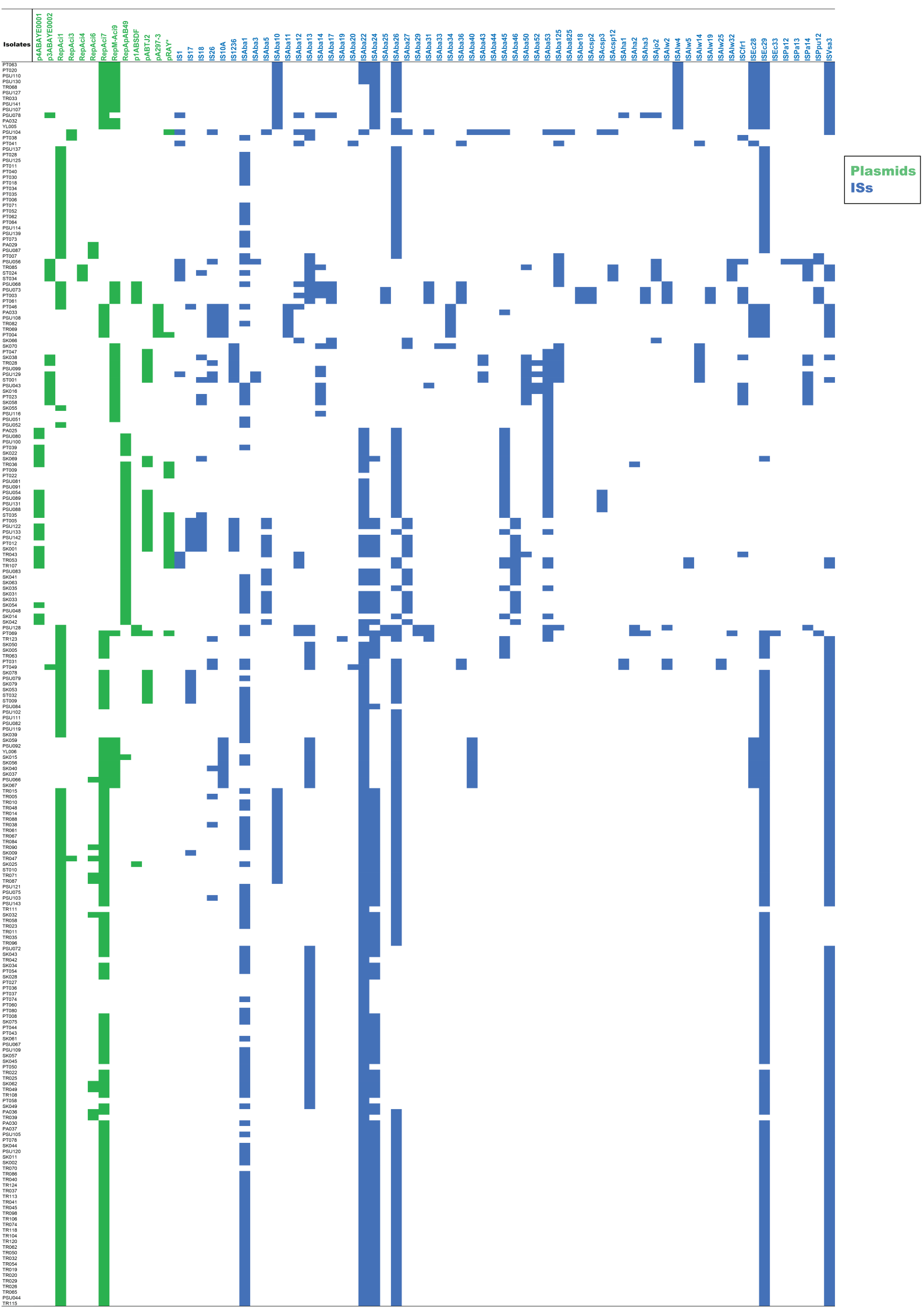

Supplement: Supplementary data 1 [file mmc1.zip › Supplementary Files/Supplementary Figures/Fig. S1.pdf]

## STs

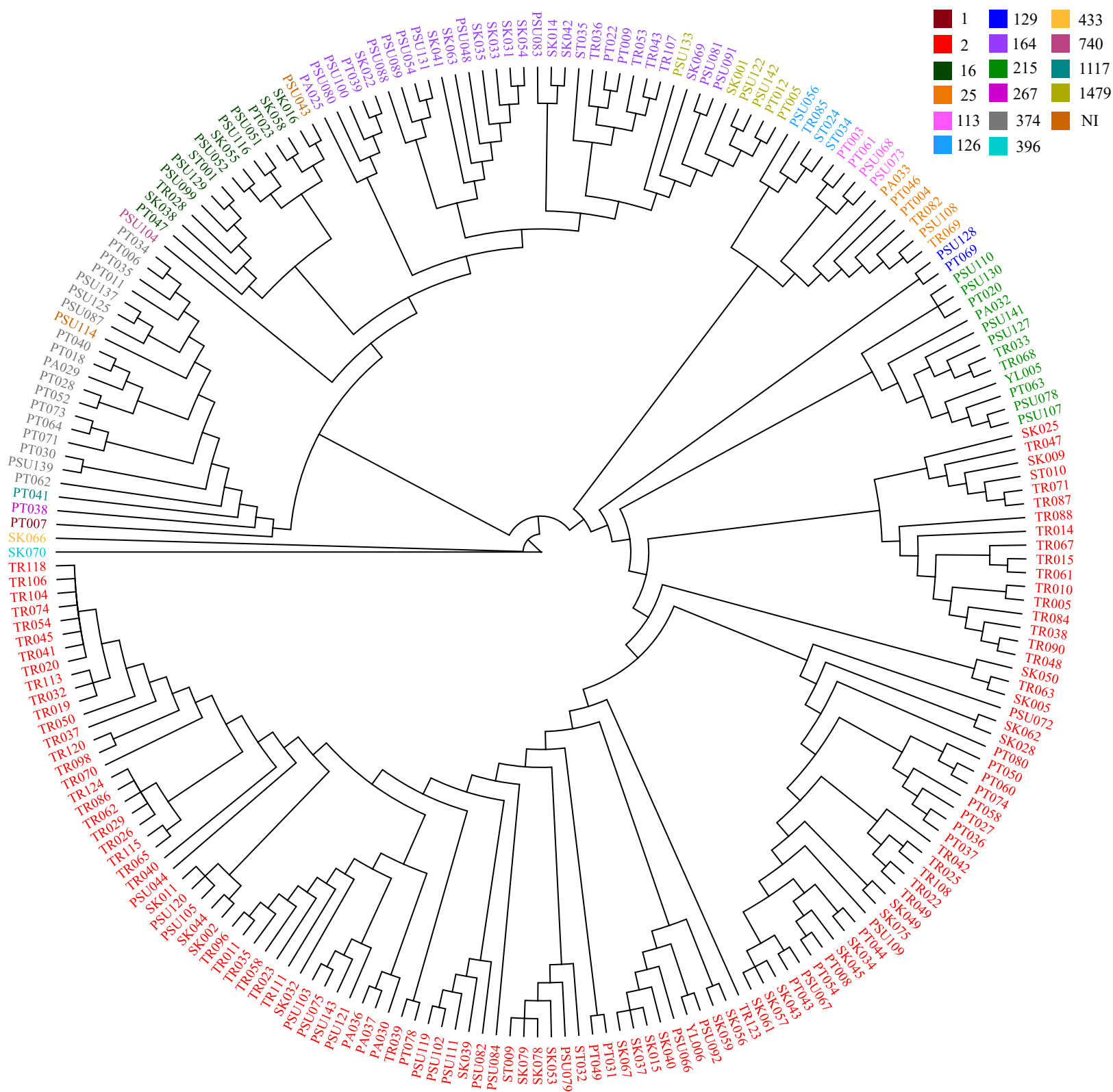

Supplement: Supplementary data 1 [file mmc1.zip › Supplementary Files/Supplementary Figures/Fig. S3.pdf]

221 isolates

Pan-genome matrix (13,567 gene clusters)

SK070  
SK066

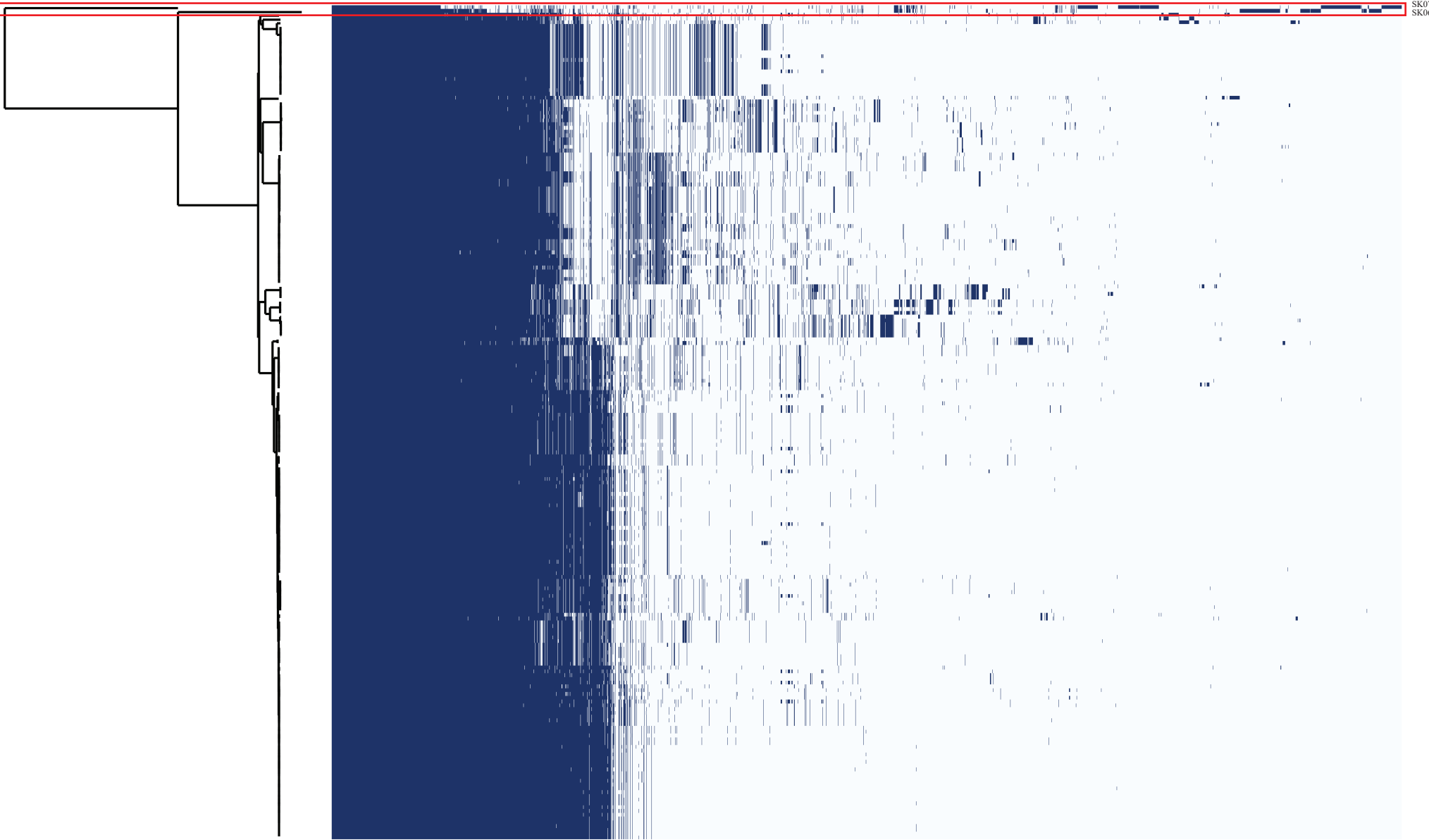

Supplement: Supplementary data 1 [file mmc1.zip › Supplementary Files/Supplementary Figures/Fig. S4.pdf]

Number of genes

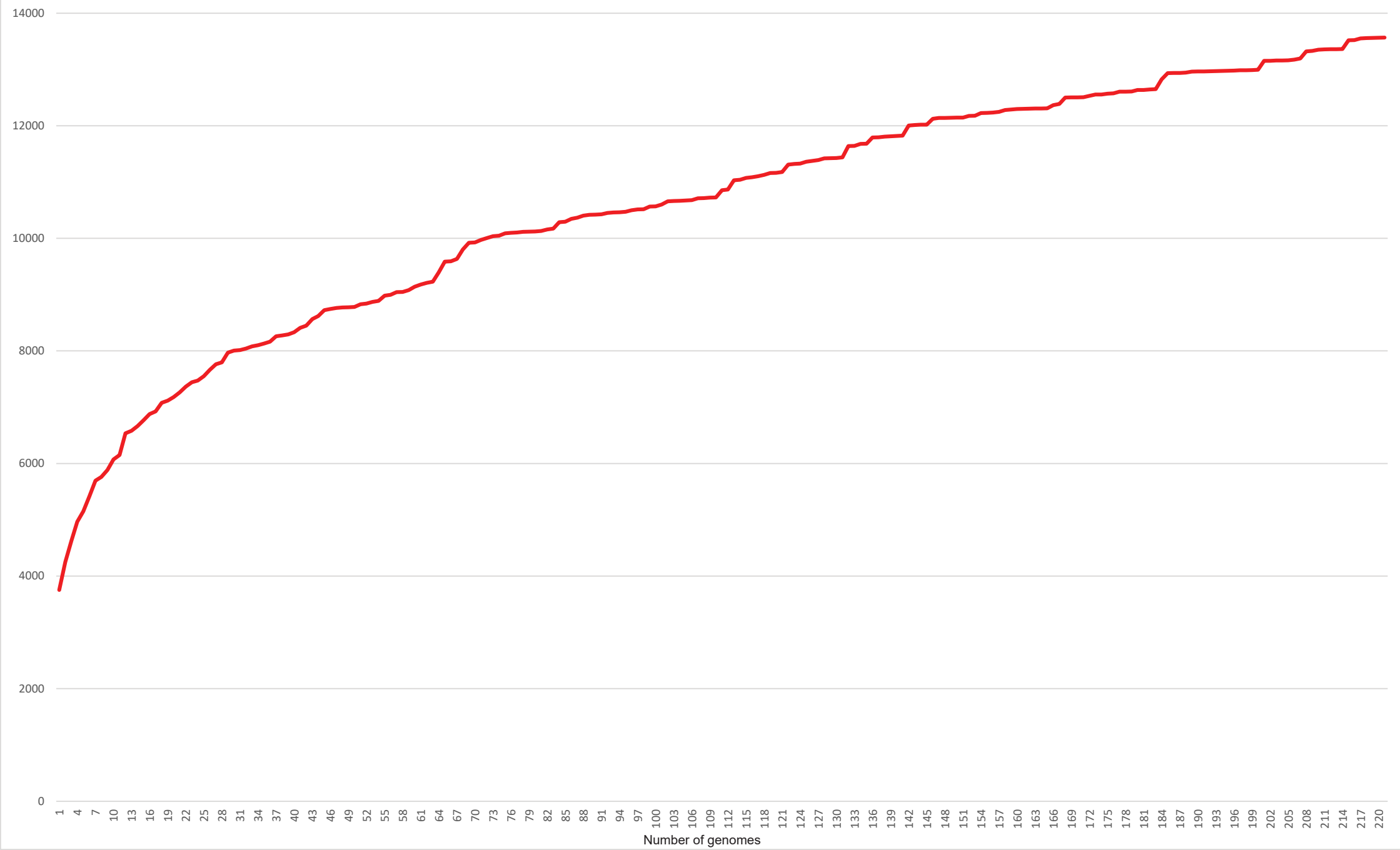

Supplement: Supplementary data 1 [file mmc1.zip › Supplementary Files/Supplementary Figures/Fig. S5.pdf]

Tree  
(409 strains)

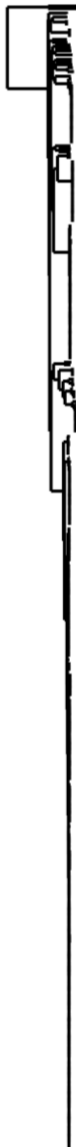

Roary matrix  
(18915 gene clusters)

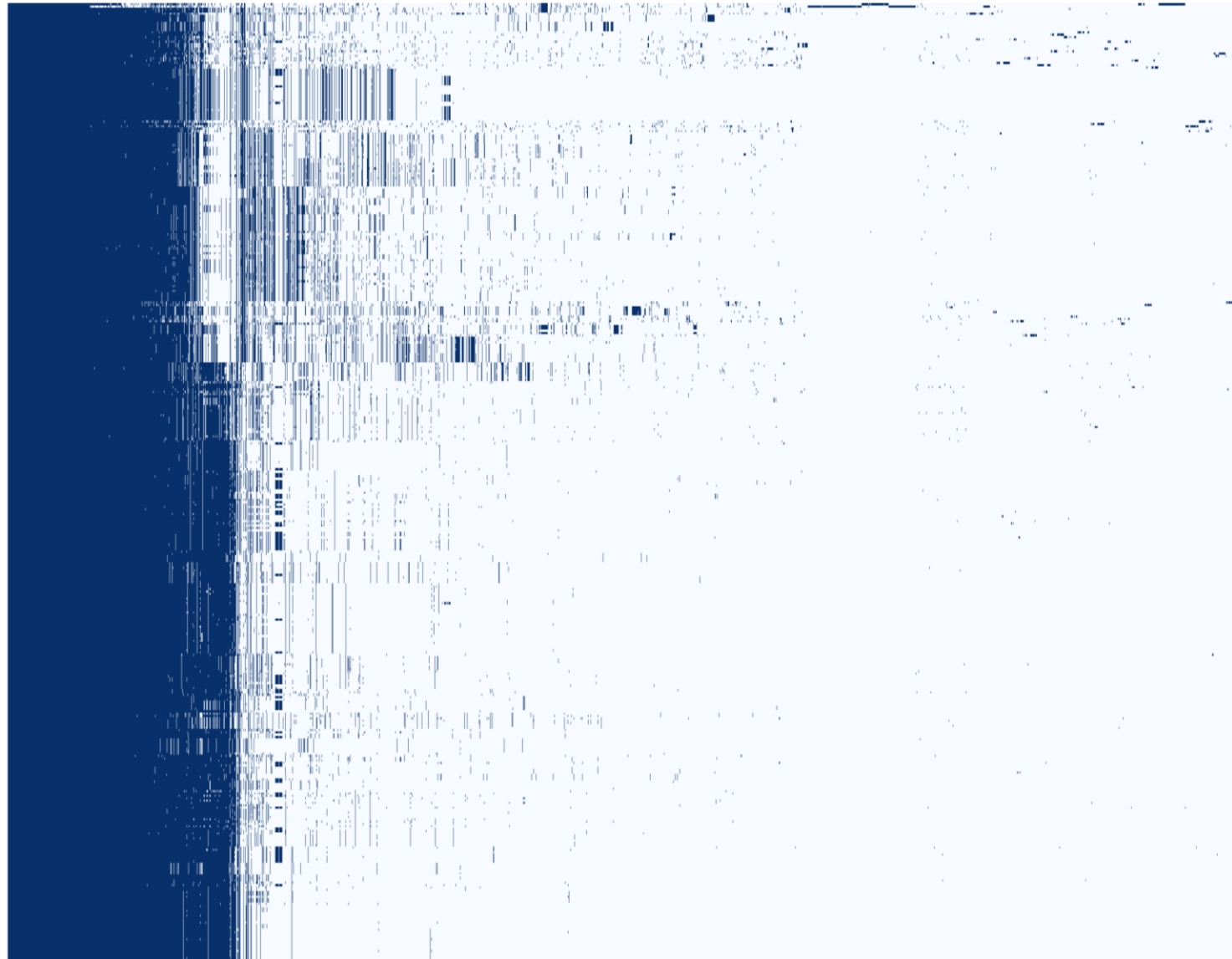

Supplement: Supplementary data 1 [file mmc1.zip › Supplementary Files/Supplementary Figures/Fig. S6.pdf]

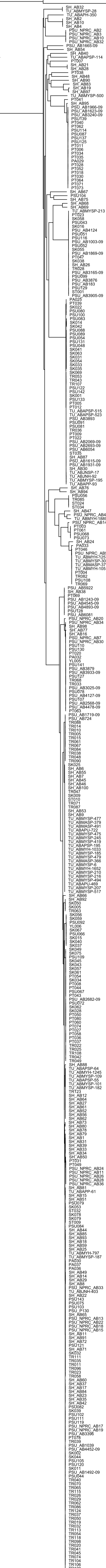

Supplement: Supplementary data 1 [file mmc1.zip › Supplementary Files/Supplementary Figures/Fig. S7.pdf]

Tree  
(409 strains)

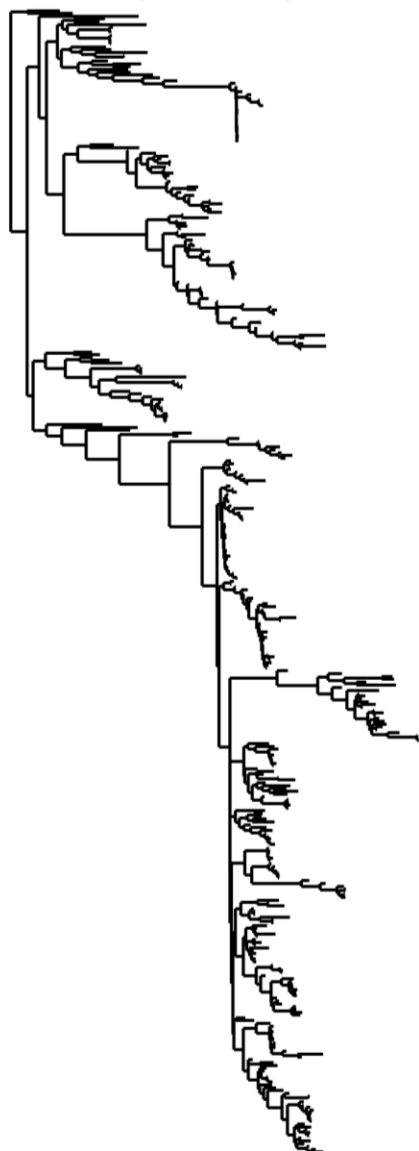

Roary matrix  
(18915 gene clusters)

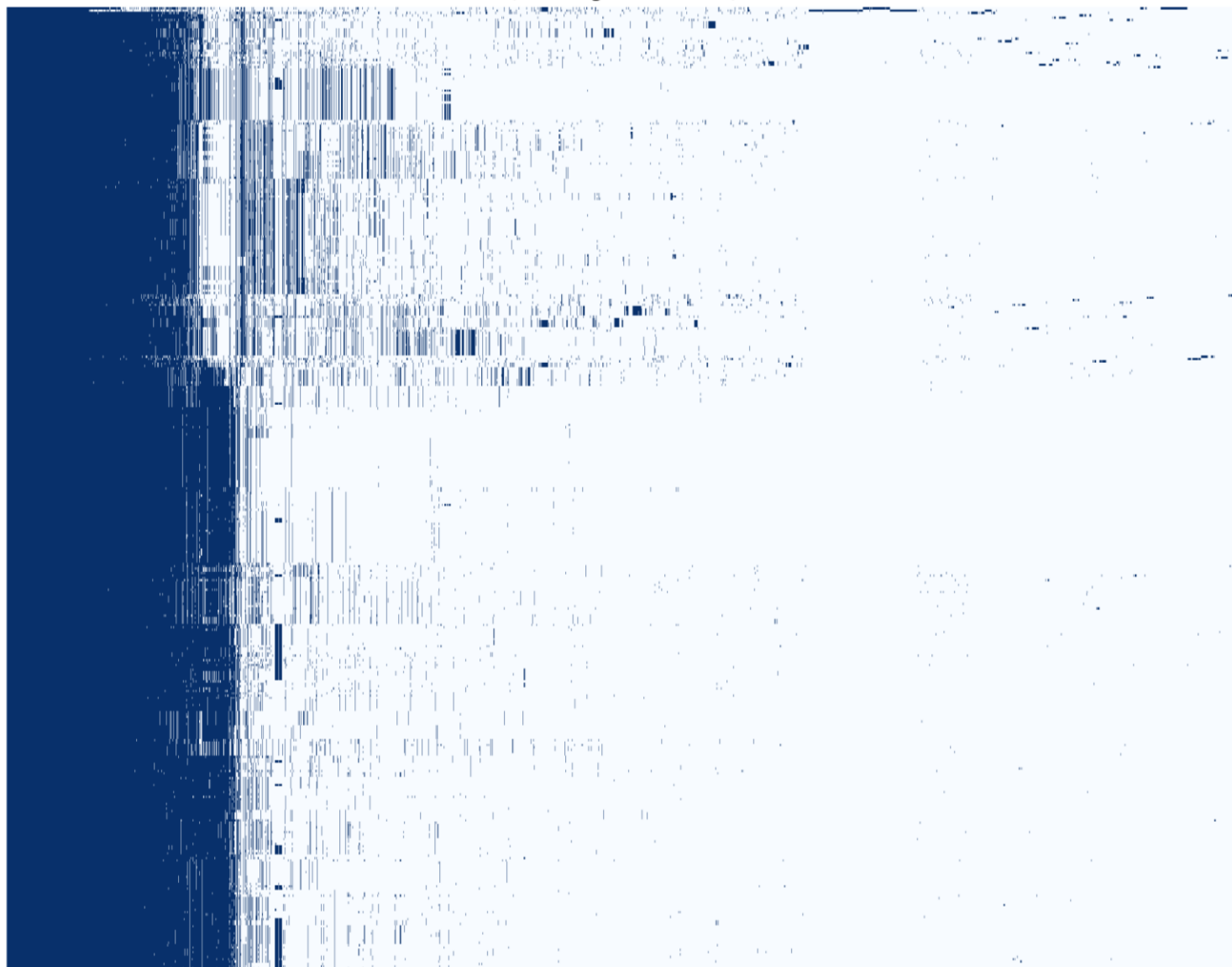

Supplement: Supplementary data 1 [file mmc1.zip › Supplementary Files/Supplementary Figures/Fig. S8.pdf]

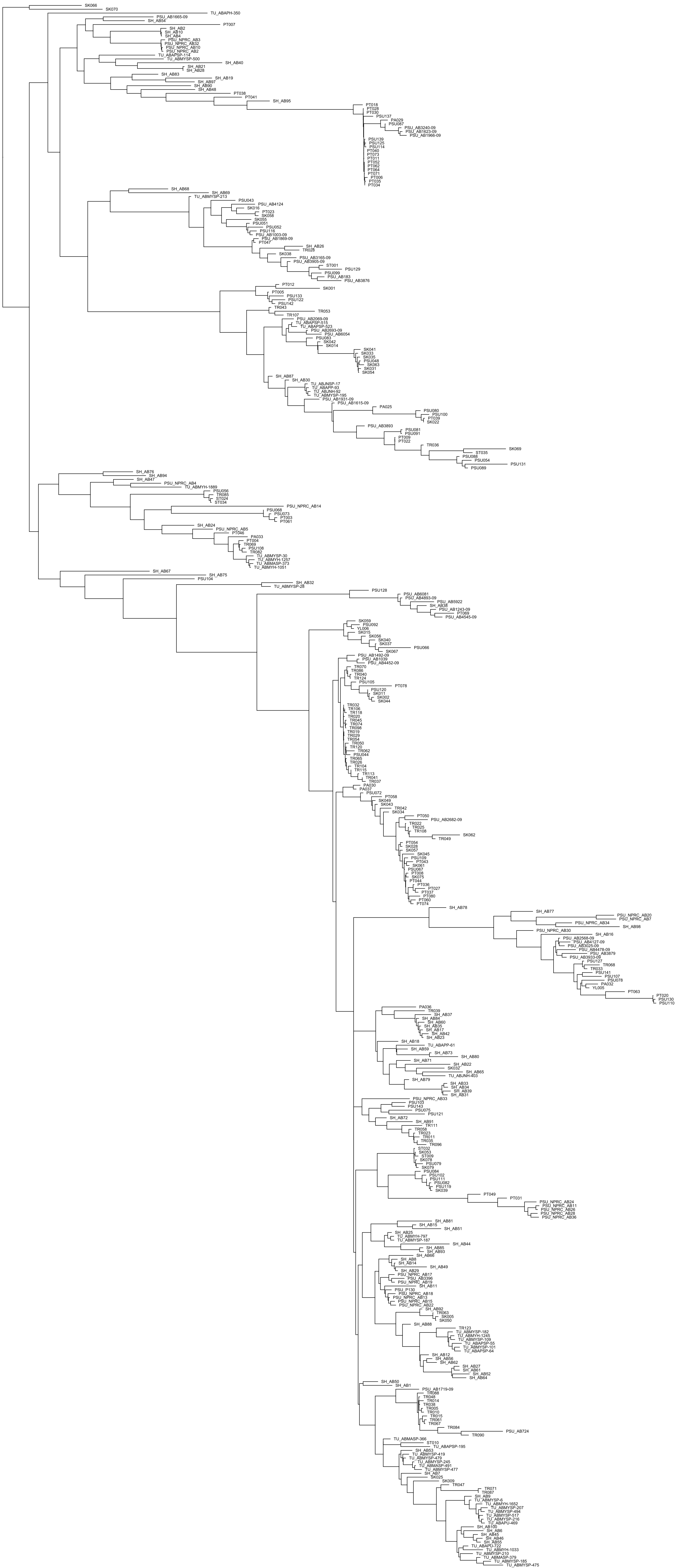

Supplement: Supplementary data 1 [file mmc1.zip › Supplementary Files/Supplementary Figures/Fig. S9.pdf]
